# Targeted PI3K/AKT-hyperactivation induces cell death in chronic lymphocytic leukemia

**DOI:** 10.1038/s41467-021-23752-2

**Published:** 2021-06-10

**Authors:** Veronika Ecker, Martina Stumpf, Lisa Brandmeier, Tanja Neumayer, Lisa Pfeuffer, Thomas Engleitner, Ingo Ringshausen, Nina Nelson, Manfred Jücker, Stefan Wanninger, Thorsten Zenz, Clemens Wendtner, Katrin Manske, Katja Steiger, Roland Rad, Markus Müschen, Jürgen Ruland, Maike Buchner

**Affiliations:** 1 Institute of Clinical Chemistry and Pathobiochemistry, School of Medicine, Technical University of Munich, Munich, Germany; 2 TranslaTUM - Central Institute for Translational Cancer Research, Technical University of Munich, Munich, Germany; 3 Institute of Molecular Oncology and Functional Genomics, TUM School of Medicine, Technical University of Munich, Munich, Germany; 4 Wellcome/MRC Cambridge Stem Cell Institute and Department of Haematology, Jeffrey Cheah Biomedical Centre, University of Cambridge, Cambridge, UK; 5 Institute of Biochemistry and Signal Transduction, Center for Experimental Medicine, University Medical Center Hamburg-Eppendorf, Hamburg, Germany; 6 Department of Medical Oncology and Hematology, University Hospital and University of Zurich, Zurich, Switzerland; 7 Munich Clinic Schwabing, Academic Teaching Hospital, Ludwig-Maximilians University (LMU), Munich, Germany; 8 Institute of Molecular Immunology, Klinikum rechts der Isar, Technische Universität München, Munich, Germany; 9 Institute of Pathology, Technische Universität München, München, Germany; 10 German Cancer Consortium (DKTK), Heidelberg, Germany; 11 Center of Molecular and Cellular Oncology, Yale Cancer Center, Yale School of Medicine, New Haven, CT USA; 12 German Center for Infection Research (DZIF), Partner Site Munich, Munich, Germany

**Keywords:** Chronic lymphocytic leukaemia, Cancer therapy

## Abstract

Current therapeutic approaches for chronic lymphocytic leukemia (CLL) focus on the suppression of oncogenic kinase signaling. Here, we test the hypothesis that targeted hyperactivation of the phosphatidylinositol-3-phosphate/AKT (PI3K/AKT)-signaling pathway may be leveraged to trigger CLL cell death. Though counterintuitive, our data show that genetic hyperactivation of PI3K/AKT-signaling or blocking the activity of the inhibitory phosphatase SH2-containing-inositol-5′-phosphatase-1 (SHIP1) induces acute cell death in CLL cells. Our mechanistic studies reveal that increased AKT activity upon inhibition of SHIP1 leads to increased mitochondrial respiration and causes excessive accumulation of reactive oxygen species (ROS), resulting in cell death in CLL with immunogenic features. Our results demonstrate that CLL cells critically depend on mechanisms to fine-tune PI3K/AKT activity, allowing sustained proliferation and survival but avoid ROS-induced cell death and suggest transient SHIP1-inhibition as an unexpectedly promising concept for CLL therapy.

## Abstract (web summary)

Current therapeutic approaches in chronic lymphocytic leukemia (CLL) focus on the suppression of PI3K/AKT signaling. Here, the authors show that CLL cells are vulnerable to hyperactivation of the PI3K/AKT signaling pathway and suggest this as a promising concept for CLL therapy.

## Introduction

Signaling derived from the B-cell surface immunoglobulin, the B-cell receptor (BCR), generally promotes the survival and proliferation of B cells. However, throughout their development, B cells are selected depending on their binding capacity to the respective antigen at several B-cell selection checkpoints. While B cells that express a functional BCR, which induces adequate BCR signaling, are positively selected by promoting B-cell survival and expansion, B cells lacking a sufficient BCR signal die of neglect^1,2^. These checkpoints also safeguard against autoimmunity by clonal deletion of autoreactive B cells that exhibit excessive BCR-signaling strength. Upon constitutively strong autoantigen binding, B cells undergo an active negative selection process that eliminates or inactivates autoreactive B-cell clones^3^. B cells are therefore selected for a narrow window of intermediate strength of BCR signaling, since both too weak (no functional BCR) and excessive strength (autoreactive BCR) of BCR signaling results in the clonal deletion and cell death. Several oncogenic drivers in B-cell malignancies constitutively activate BCR signaling and thereby mimic signaling that promotes positive selection and B-cell expansion^4^.

In chronic lymphocytic leukemia (CLL), autonomous autoreactive BCR signaling contributes to tumor cell survival^5^, and established targeted therapy concepts focus on inhibiting oncogenic kinases in the BCR pathway^6–8^, resulting in signal deprivation and thereby cell death^9,10^. However, despite initial remission of CLL, patients frequently relapse with refractory disease or eventually progress to Richter syndrome transformation with limited treatment options^11^. Recent evidence in pre-B-cell acute lymphoblastic leukemia (pre-B ALL) suggests that targeted hyperactivation of signaling components downstream of the BCR above a maximum threshold will also invariably trigger cell death^12–14^. This indicates that, despite the transformation, malignant B cells remain vulnerable to checkpoint signals for removal of autoreactive clones. While direct agonists of BCR-downstream kinases are not available, targeted hyperactivation can be achieved by pharmacological inhibition of negative regulators of the BCR-signaling pathway (e.g., inhibitory phosphatases including SHIP1), resulting in the activation of signaling above a maximum tolerable threshold causing energy stress and cell death. This effect likely occurs because pre-B ALL cells are in an early developmental B-cell stage, where B cells are subject to central tolerance checkpoints for removal of autoreactive clones^15^. As exemplified in pre-B ALL, pharmacological hyperactivation of BCR signaling may represent a powerful strategy to overcome conventional drug resistance and to prevent relapse induced by long-term kinase inhibitor treatment.

While in many B-cell malignancies, the activation of the BCR-signaling pathway occurs via genetic activation of signaling mediators^16,17^ or by BCR mimicry^18,19^, the BCRs expressed by CLL cells frequently recognize autoantigens, including an internal epitope of their own BCR^5^. The activation of the BCR-signaling pathway occurs primarily in secondary lymphoid organs^20^. Here, it typically converges with activation of the phosphatidylinositol-3-kinase (PI3K) signaling pathway downstream of homing chemokine receptors and adhesion molecules^21,22^, with the PI3Kδ isoform as the most prevalent PI3K subclass in mature B cells^23^. Upon its recruitment and activation by binding to phosphorylated YxxM motifs in membrane coreceptors, such as CD19 or BCAP in case of BCR stimulation, PI3K then phosphorylates the lipid phosphatidylinositol 4,5-bisphosphate [PI(4,5)P_2_] to generate phosphatidylinositol-3,4,5-trisphosphate [PI(3,4,5)P_3_], which acts as a pivotal second messenger signaling molecule by providing a binding site for intracellular enzymes that contain pleckstrin homology (PH) domains. For instance, AKT requires binding with its PH domain to PI(3,4,5)P_3_ to become enzymatically active. The AKT1 isoform in CLL cells plays an important role in driving cell proliferation, growth, survival, and cellular metabolism^24,25^.

The PI3K signaling is negatively regulated by the SH2-containing inositol 5′-phosphatase SHIP1 that hydrolyzes PI(3,4,5)P_3_ to PI(3,4)P_2_, thereby preventing the recruitment and activation of PH domain-containing effectors and the propagation of PI3K-mediated downstream signals^26^. Mutations causing hyperactivation of the PI3K pathway are among the most common genetic lesions in human cancer^27^, and somatic mutations in the *INPP5D* gene encoding SHIP1 have been detected in acute myeloid leukemia (AML) patients^28^. These mutations strongly reduce SHIP1 activity, either by directly interfering with the enzymatic activity of SHIP1 to suppress PI3K/AKT signaling, or by loss of function of their SH2 domain or PXXP motifs, both of which are required for proper recruitment of the inhibitory complex^29^. Similarly, frame-shifts, as well as other translationally-inactivating deletions and insertions in the *INPP5D* gene, occur in T-cell acute lymphoblastic leukemia (T-ALL)^30^. In strong contrast, SHIP1 inhibition or genetic deletion of *INPP5D* in BCR-ABL1-driven pre-B ALL mimics excessively strong signaling from an autoreactive BCR and engages a B-cell intrinsic negative selection program leading to energy stress and cell death^12,14^. Pre-B ALL cells are derived from B-cell precursors that are subject to central B-cell tolerance checkpoints. CLL cells exhibit a mature B-cell phenotype and are thought to be derived from naïve B cells. Here we tested whether CLL cells—like pre-B ALL cells—are subject to mechanisms of negative B-cell selection. Previous studies have suggested that SHIP1 is expressed in CLL;^31^ however, the functional role of SHIP1 in limiting PI3K signaling in established CLL is still not clearly defined.

In this study, we, therefore, investigated the cellular consequences of acute AKT activation and SHIP1 inhibition in CLL in vitro and in vivo. While intermediate levels of PI3K/AKT activity are essential for the survival of CLL cells, we show that the negative regulator SHIP1 is required to balance PI3K/AKT signaling in CLL to prevent hyperactivation of BCR-downstream signaling and clonal deletion. Accordingly, we propose transient SHIP1 phosphatase inhibition as a potential therapeutic option and a promising strategy to overcome mechanisms of drug resistance in CLL.

## Results

### Acute AKT1 hyperactivation in CLL is detrimental to CLL cells

**Fig. 1:**
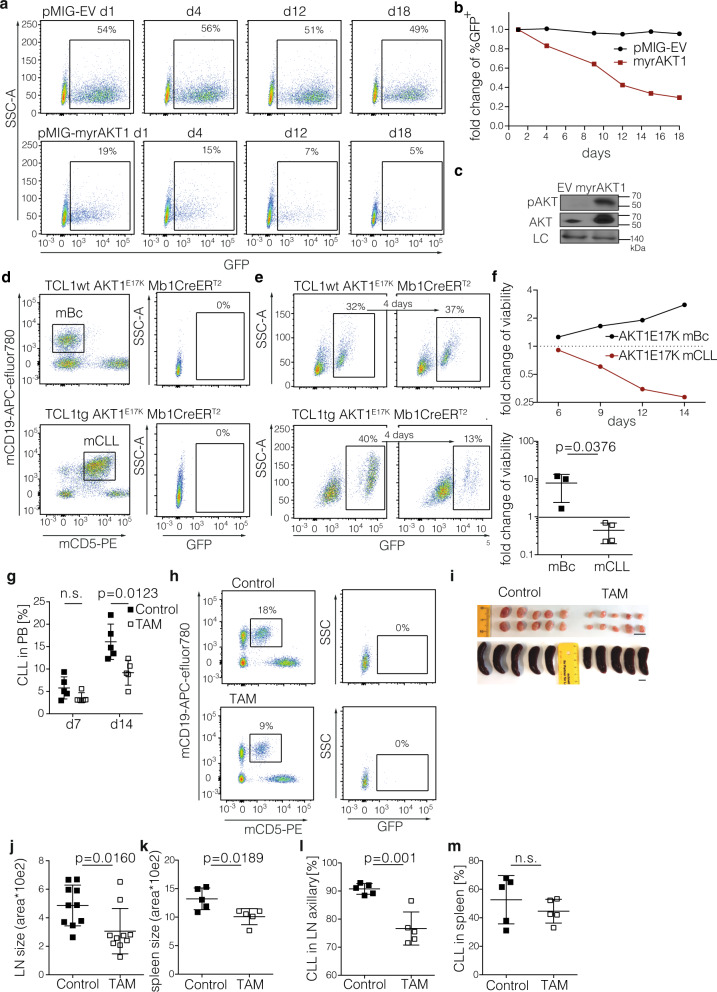
Acute AKT1 activation is detrimental to CLL cells of murine and human origin. **a** Time course FACS analysis of GFP expression in MEC-1 cells upon transduction with pMIG EV (empty vector) or pMIG-myrAKT1, representative for 2 independent experiments. The gating strategies are shown in Supplementary Fig. S5. **b** Fold change of GFP expression in MEC-1 Eco cells upon transduction with pMIG-EV (black squares) or pMIG-myrAKT1 (red squares), pooled analysis of 2 independent experiments. **c** Immunoblot confirmation of active AKT expression in GFP-sorted MEC-1 Eco cells upon transduction with pMIG-EV or pMIG-myrAKT1, representative for 2 independent analyses. DHX9 serves as a loading control (LC). **d** Splenocytes from aged TCL1wt AKT1^E17K^ Mb1-CreER^T2^ (top) or TCL1tg AKT1^E17K^ Mb1-CreER^T2^ (bottom) mice were analyzed for CD19 and CD5 expression (CLL development). GFP negativity of murine B cells (mBc) in the upper panel and murine CLL (mCLL) in the lower panel was confirmed (to exclude potential leakiness of the CreER^T2^ system). Representative data for *n* = 3 (CLL) and *n* = 2 (mBc) mice. **e** GFP expression was confirmed in both normal B cells (upper panel, GFP blot is gated for viable CD19^+^CD5^−^ cells) and CLL cells (lower panel, GFP blot is gated for viable CD19^+^CD5^+^ cells) upon TAT-Cre or 4-OHT administration, indicating AKT1^E17K^ transgene expression (left panels). 4 days later, GFP^+^ normal murine B cells (mBc; upper panel) were slightly enriched while GFP^+^ murine CLL cells (mCLL; lower panel) significantly declined (right panels) over time. Representative data for *n* = 3 (mCLL) and *n* = 2 (mBc) mice. **f** The differential response to AKT1^E17K^ expression on the viability of mBc (black squares) and mCLL (red squares) by induction of AKT1^E17K^ on cell viability is shown over time (representative data are shown in top panel) and a summary of three independent experiments from *n* = 3 (CLL) and *n* = 2 (mBc) biologically independent mice (d4-8, depending on TAT-Cre or 4-OHT induction; lower panel). Data are presented as individual values and mean values ± standard deviation (SD). Statistical significance was assessed by a two-tailed unpaired Student’s t-test. **g**–**m** Splenocytes from an aged TCL1tg AKT1^E17K^ Mb1-CreER^T2^ mouse were transplanted into 10 *wt* recipients and provided with tamoxifen-containing chow (TAM; clear squares; *n* = 5 individual mice) or control chow (control; black squares; *n* = 5 individual mice) for 4 weeks, thereafter all mice received control chow. On d7 and d14, CLL content was monitored in the peripheral blood by flow cytometric analysis of CD19^+^CD5^+^ cells. Data are presented as individual values and mean values ± SD, statistical significance was assessed by a two-tailed unpaired Student’s *t*-test; (n.s.) not significant (**g**). Representative example of GFP analysis for CLL in the peripheral blood (PB) on d14 post injection and tamoxifen (TAM) or control chow administration (**h**). Macroscopic analysis of lymph nodes (axillary) and spleens 8 weeks post transplantation. Scale bars indicate 1 cm. (**i**). Lymph node (**j**) and spleen size (**k**) are shown, determined by area quantification using the ImageJ software. Percent CD19^+^CD5^+^ CLL cells in the lymph nodes (**l**) and spleen (**m**) of *wt* mice 8 weeks post transplantation is shown (**j**), for *n* = 5 individual animals, respectively. Data are presented as individual values and mean values ± SD and statistical significance was assessed by a two-tailed unpaired Student’s *t*-test in **j**–**m**; Source data are provided as a Source Data file.

In order to investigate how CLL cells respond to acute activation of the PI3K/AKT signaling pathway, we transduced the CLL-derived cell line MEC-1 with a constitutively active form of AKT1 (myrAKT1^32^) linked with GFP. Unexpectedly, we observed a decrease in myrAKT1-expressing GFP^+^ MEC-1 cells over time as compared to the untransduced, GFP-negative cells while the fraction of GFP empty vector (EV) control cells remained stable in the culture (Fig. 1a, b). AKT overexpression and activation was confirmed using western blot in myrAKT1-expressing MEC-1 cells as compared to EV transduced cells sorted for GFP (Fig. 1c). These results indicate that acute activation of the PI3K/AKT signaling pathway diminishes CLL cell viability and expansion in this model and thereby contradict the current understanding of a solely proliferative and prosurvival role of AKT1 activation in CLL.

To extend these findings in primary CLL cells, we employed the TCL1 transgenic (TCL1tg) CLL mouse model^33^. For this, we first created a transgenic mouse line for inducible AKT1^E17K^ expression, a mutation derived from solid tumors and T-cell lymphoma^34^ that renders AKT1 constitutively active by increasing the affinity for PI(3,4,5)P_3_ and confering affinity to the abundant plasma membrane lipid PI(4,5)P_2_, which is not bound by AKT1 under normal circumstances^35^. We introduced the human AKT1^E17K^ cDNA preceded by a loxP-flanked transcriptional and translational STOP cassette into the ubiquitously expressed Rosa26 locus, followed by an IRES GFP cassette to track AKT1^E17K^-expressing cells^36^ (Supplementary Fig. 1a–c). The resulting Rosa26^loxSTOPlox^AKT1^E17K^ mice were crossed with Mb1-CreER^T2^ transgenic animals for B-cell–specific inducible Cre expression^37^. The STOP cassette is excised upon tamoxifen (4-OHT) treatment specifically in the B-cell-lineage, and AKT1^E17K^ expression is indicated by GFP expression. After crossing the AKT1^E17K^ Mb1-CreER^T2^ line to the TCL1tg CLL model, we obtained triple transgenic animals carrying TCL1tg AKT1^E17K^ Mb1-CreER^T2^ and waited for CLL development in these mice without activating the AKT1^E17K^ transgene. Once signs of disease occurred, we harvested splenocytes of the TCL1tg AKT1^E17K^ Mb1-CreER^T2^ mice and confirmed CLL development by detecting a pronounced CD19^+^CD5^+^ CLL cell population in the spleen that did not express the AKT1 transgene, as indicated by the GFP-negative CLL/B-cell population (Fig. 1d). As a control for normal, non-transformed B cells, we isolated splenocytes from a littermate carrying the AKT1^E17K^ transgene and Mb1-CreER^T2^ but not the TCL1 transgene (TCL1wt). We then tested for the AKT1^E17K^/GFP expression by administering 4-OHT in vitro. While both non-transformed B cells and CLL cells recombined the locus and expressed AKT1^E17K^ as indicated by GFP expression, AKT1^E17K^ promoted B-cell survival only in non-transformed B cells in vitro, while the GFP^+^ CLL cells rapidly decreased and died in vitro (Fig. 1e, f).

Next, we investigated whether the inducible activation of AKT1^E17K^ affects murine CLL growth in vivo. To this end, we transplanted splenocytes of aged TCL1tg AKT1^E17K^ Mb1-CreER^T2^ mice into wild-type (*wt)* recipients and induced AKT1^E17K^ expression by providing tamoxifen-enriched chow or control chow for 4 weeks. We followed the CLL content in the peripheral blood and observed a significant drop in the CLL content of the tamoxifen-treated group post transplantation (Fig. 1g). However, no GFP^+^ CLL cells were detected in the peripheral blood suggesting that AKT1^E17K^ expression is not tolerated in murine CLL cells as dying cells are rapidly cleared and therefore not detectable in vivo^38^ (Fig. 1h). To exclude the possibility that AKT1^E17K^-expressing CLL cells relocate to the secondary lymphoid organs, we harvested spleens and lymph nodes 8 weeks post transplantation. We found a moderate decrease in the spleen size as well as decreased lymph node sizes in the tamoxifen-treated group as compared to the control mice (Fig. 1i–k). When we performed flow cytometric analyses for CD19, CD5, and GFP expression, we observed a moderate reduction in CLL in the lymph nodes (Fig. 1l) but not in the spleen (Fig. 1m). Importantly, we did not detect a substantial percentage of GFP^+^ CLL cells in the lymphoid organs despite tamoxifen treatment. Therefore, the vast majority of CLL in the tamoxifen-treated mice did not express GFP/AKT1^E17K^, suggesting that virtually all CLL cells that recombined the locus to express AKT1^E17K^ had died and were cleared in vivo^38^, which is in line with our in vitro studies. However, there was a small but clearly visible GFP^+^ population detectable in the peritoneal cavity and the lymph node of tamoxifen-treated mice (less than 0.2% of CLL cells, summarized in Supplementary Fig. 1d, e) but not in the control mice, demonstrating that the genetic rearrangement had occurred upon tamoxifen treatment but did not result in CLL cell expansion. Taken together, we, therefore, conclude that acute hyperactivation of the PI3K/AKT pathway by introducing constitutively active AKT1 in CLL is detrimental for the cells in vitro and in vivo.

### The inhibitory phosphatase SHIP1 is highly expressed and active in CLL

**Fig. 2:**
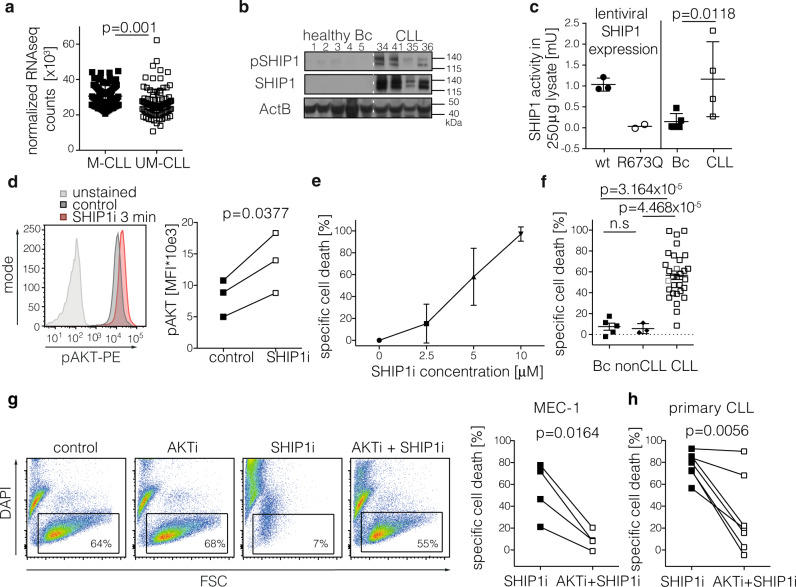
SHIP1 activity in CLL samples is required to limit AKT signaling for CLL cell survival. **a** Normalized RNAseq counts of *INPP5D* mRNA (encoding SHIP1) are shown for IgV_H_-mutated CLL cases (M-CLL; black squares; *n* = 94) as compared to IgV_H_-unmutated CLL samples (UM-CLL; clear squares; *n* = 95); samples obtained from the peripheral blood. Data are presented as individual values and mean values ± SD. Statistical significance was assessed by a two-tailed unpaired Student’s *t*-test. **b** Immunoblot of pSHIP1^Y1020^ and global SHIP1 with beta-actin (ActB) as a loading control in MACS-isolated CD19^+^ peripheral blood B cells from healthy donors (*n* = 5) as compared to CLL samples obtained from peripheral blood (*n* = 4). Numbers indicated reflect the sample IDs of the CLL samples listed in Supplementary Table 1. **c** Direct phosphatase activity was determined using Malachite green assay after pulldown from 250 μg protein isolated from (left) H1299 cells lentivirally overexpressing *wt* SHIP1 or the AML-derived R673Q variant of SHIP1 (as positive and negative control, respectively) and (right) healthy donor peripheral blood B cells (*n* = 5) or primary peripheral blood CLL cells (*n* = 4). Data are presented as individual values and mean values ± SD. Statistical significance was assessed by a two-tailed unpaired Student’s *t*-test. The respective protein expression is shown in Supplementary Fig. S2c. **d** MEC-1 cells were treated with the SHIP1 inhibitor (SHIP1i) 3AC 5 μM (red line) or vehicle (dark gray line) for 3 min and subjected to intracellular pAKT staining; unstained control cells are indicated in light gray; representative FACS blot (left) and the summary of three independent experiments (right) is shown. Right: Data are presented as individual values for each experiment; statistical significance was assessed by a two-tailed paired Student’s *t*-test. **e** Cytotoxicity dose–response to increasing concentrations of the SHIP1 inhibitor 3AC in 28 primary CLL samples. The percentage of dead cells was determined by flow cytometry via DAPI staining. The percentage of specific cell death was calculated as follows: 100 × (% dead cells − % baseline dead cells)/(100% − % baseline dead cells). Data are presented as mean values ± SD. **f** Viability was determined upon 48 h treatment with 5 μM 3AC in vitro in CD19^+^ B cells from healthy donors (filled squares), the B-cell lymphoma lines BJAB (human Burkitt lymphoma), SUDHL6 (human diffuse, mixed small and large cell lymphoma line), Bal17 (murine B-cell lymphoma; filled diamonds), and 28 primary CLL samples (clear squares) as well as the CLL-derived cell lines MEC-1 (light gray square) and EHEB (dark gray square), and the specific cell death was calculated as described in **e**. Data are presented as individual values and mean values ± SD. Statistical significance was assessed by a two-tailed unpaired Student’s *t*-test. **g** MEC-1 cells were treated with vehicle (control), treated with the AKT inhibitor AZD-5363 (5 μM) alone, or in combination with 3AC (5 μM). Left: representative flow cytometry analysis of DAPI negative, viable cells upon the treatments. Right: the specific cell death was determined as described in **e**, measured in four independent experiments. Data are presented as individual values for each experiment and statistical significance was assessed by a two-tailed paired Student’s *t*-test. **h** Primary CLL cells derived from the peripheral blood of 7 donors were treated with vehicle (control), treated with the AKT inhibitor AZD-5363 (5 μM) alone or in combination with 3AC (5 μM) and the specific cell death was determined as described in **e**. Data are presented as individual values per CLL donor and statistical significance was assessed by a two-tailed paired Student’s *t*-test. Source data are provided as a Source Data file.

We next identified strategies to therapeutically exploit the sensitivity of CLL cells to PI3K/AKT hyperactivation. As direct activators of PI3-kinases for pharmacological studies are not available, we investigated whether targeted hyperactivation can be achieved by pharmacologically inhibiting the inhibitory phosphatase SHIP1. To determine whether SHIP1 inhibition could present a useful approach in CLL, we first investigated SHIP1 expression and activity in CLL samples. The analysis of mRNA expression levels of *INPP5D*, encoding the phosphatase SHIP1, revealed that all 210 CLL samples analyzed^39^ expressed SHIP1 mRNA, with higher levels in samples derived from CLL patients with mutated IgV_H_ genes that have a favorable clinical prognosis as compared to those with an unmutated IgV_H_ (Fig. 2a). However, there is no significant association of SHIP1 mRNA expression with CLL patients’ time to treatment or overall survival (Supplementary Fig. 2a). We then analyzed SHIP1 protein levels of primary CLL samples and compared them to B cells of healthy age-matched donors, both derived from the peripheral blood, and found significantly higher SHIP1 expression and phosphorylation levels in CLL than in normal peripheral B cells, which is potentially induced by the activated BCR status in CLL^5^ (Fig. 2b). CLL patients’ characteristics for all samples used in this study are listed in Supplementary Table 1. In AML, recurrent mutations in the SHIP1-encoding gene *INPP5D* lead to a significantly reduced phosphatase activity^29^. To determine whether recurrent loss-of-function mutations in the SHIP1-encoding *INPP5D* gene also exist in CLL, we next identified the types and frequency of *INPP5D* alterations in CLL in publically available sequencing data^40–42^ (using the cBioportal for Cancer Genomics platform (http://cbioportal.org)) and compared them to *INPP5D* mutations previously found to reduce SHIP1 phosphatase activity^29^ or protein stability^43^. In total, only 0.6% of CLL samples analyzed carried *INPP5D* gene alterations in CLL (6 mutations detected in 1048 analyzed CLL samples). Of the 6 mutations, none occurred recurrently or was previously described as inactivating the SHIP1 phosphatase activity (listed in Supplementary Fig. 2b). Thus, *INPP5D* gene alterations are rare in CLL suggesting that SHIP1 is enzymatically active in the vast majority of CLL cases. To further confirm that the enzymatic activity of SHIP1 was stable in primary CLL samples, we performed a malachite green phosphate assay after SHIP1 precipitation. In primary CLL samples, we found variable but detectable levels of PI(3,4,5)P_3_ dephosphorylating activity in all samples comparable to control cells that lentivirally overexpressed *wt* SHIP1. In healthy donor B-cell samples, SHIP1 activity was below the detection limit in 3 out of 5 tested donors, possibly due to the low expression levels in the absence of additional stimuli (Fig. 2c and Supplementary Fig. 2c). Taken together, we, therefore, conclude that CLL cells express high levels of enzymatically active SHIP1.

### SHIP1 inhibition induces AKT activation and is toxic specifically for CLL cells

To investigate the functional relevance of SHIP1 phosphatase activity in CLL, we first tested a small molecule SHIP1 inhibitor 3AC (3 α-Aminocholestane) that selectively inhibits the enzymatic activity of SHIP1 (IC_50_ ~2.5 μM) but not related phosphatases SHIP2 and PTEN (IC_50_ > 1 mM)^44^. To confirm that SHIP1 inhibition hyperactivates the PI3K/AKT signaling pathway in CLL, we analyzed AKT S473-phosphorylation levels and, as expected^45^, found significantly increased activation upon SHIP1 inhibition (Fig. 2d). Further analyses of downstream events indicated the transient activation of the mTOR/S6 signaling pathway upon 3AC treatment with upregulation of the anti-apoptotic protein MCL1, followed by a decrease below baseline levels (Supplementary Fig. 2d). At continuous treatment for 48 h, the SHIP1 inhibitor induced dose-dependent cell death in all 28 primary CLL samples tested (Fig. 2e). To account for the variation in the vehicle-treated control cells due to spontaneous apoptosis of primary CLL cells in vitro, we calculated the specific cell death induced by 3AC^46^. In order to determine whether the cytotoxic effects were specific for CLL, we compared the effects of 3AC on the viability of non-malignant B cells purified from the peripheral blood of healthy donors, as well as several B-cell lymphoma cell lines, namely BJAB, SUDHL6, and Bal17, to primary CLL samples and the CLL-like cell lines MEC-1 and EHEB. While B cells derived from the peripheral blood of healthy donors and Bal17, BJAB and SUDHL6 lymphoma cells remained largely unaffected in terms of viability upon 3AC treatment (Fig. 2f), SHIP1 inhibition-induced cell death specifically in CLL cells, including the MEC-1 and EHEB cell lines. Amongst CLL samples, cells derived from patients with a favorable prognosis (mutated IgV_H_) were more sensitive to SHIP1 inhibition than those with a poor prognosis (expressing unmutated IgV_H_; Supplementary Fig. 2e). In order to further clarify whether the activation of AKT is critical for 3AC-mediated cytotoxicity, we treated MEC-1 cells with the AKT inhibitor AZD-5363 and evaluated cell viability upon SHIP1 inhibition. Strikingly, co-treatment with the AKT inhibitor significantly reduced the cytotoxic effect of the SHIP1 inhibitor 3AC (Fig. 2g), while AKT inhibition alone had no significant effect on cell viability (Supplementary Fig. 2f). This was also confirmed in samples derived from the peripheral blood of seven individual CLL patients (Fig. 2h). Together, this data demonstrates that SHIP1 inhibition mediates cytotoxicity specifically in CLL via AKT activation.

### SHIP1 inhibition delays CLL progression in vivo

**Fig. 3:**
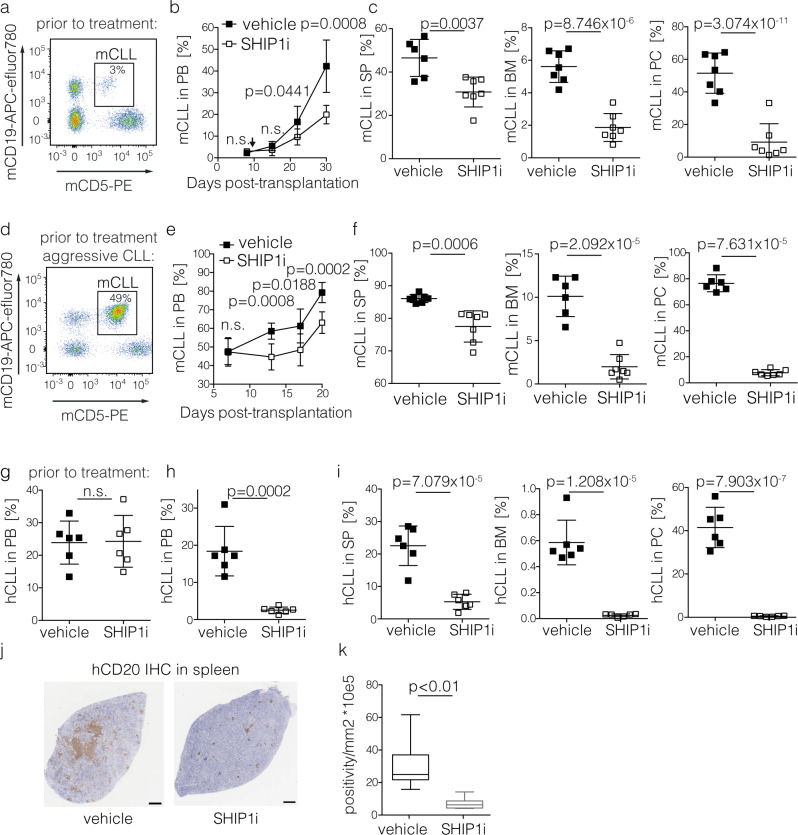
SHIP1 Inhibition reduces CLL progression in vivo. **a** Representative FACS analysis of murine CLL cells (mCLL) in the peripheral blood prior to treatment initiation (d8). **b** Time course of mCLL content in the peripheral blood (PB), arrow indicates the SHIP1 inhibitor (SHIP1i) 3AC (clear squares) or vehicle (black squares) treatment initiation (*n* = 7 individual animals per treatment group, representative for two independent experiments), with 14 total doses of 20 mg/kg (treatment schedule is shown in Supplementary Fig. S3a). Data are presented as mean values ± SD. Statistical significance was assessed by a two-tailed unpaired Student’s *t*-test for the indicated time points. **c** murine CLL cell engraftment in the spleen (SP), the bone marrow (BM) and in peritoneal cavity (PC) after treatment with 3AC or vehicle control is shown as determined by FACS analysis; *n* = 7 animals per treatment group, representative for two independent experiments. Data are presented as individual values and mean values ± SD. Statistical significance was assessed by a two-tailed unpaired Student’s *t*-test. **d** Representative FACS analysis of aggressive mCLL cells in the peripheral blood prior to treatment initiation (d10). **e** Time course of mCLL content in the peripheral blood, arrow indicates 3AC/vehicle treatment initiation (*n* = 7 animals per treatment group; representative for two independent experiments; treatment schedule is shown in Supplementary Fig. S3b). Data are presented as mean values ± SD. Statistical significance was assessed by a two-tailed unpaired Student’s *t*-test for the indicated time points. **f** Murine CLL cell engraftment in the spleen (SP), the bone marrow (BM), and in peritoneal cavity (PC) after treatment with the SHIP1i 3AC or vehicle control is shown as determined by FACS analysis. Data are presented as individual values and mean values ± SD. Statistical significance was assessed by a two-tailed unpaired Student’s *t*-test. **g** Summary of human CLL (hCLL) contents in the peripheral blood prior to treatment initiation (d1 post injection), representative for two independent experiments. **h**–**j** Reduction of CLL cells in the peripheral blood (**h**), and spleen, bone marrow, and peritoneal cavity (**i**) of NSG mice treated with 3AC (*n* = 6) as compared to vehicle control (*n* = 6), representative for two independent experiments. The treatment schedule is shown in Supplementary Fig. S3c and the gating strategy in Supplementary Fig. S3d. Data are presented as individual values and mean values ± SD. Statistical significance was assessed by a two-tailed unpaired Student’s *t*-test. Representative result for two independent CLL donors injected in 12 NSG mice, respectively. **j**–**k** Immunohistochemistry (IHC) for human CD20 (hCD20) in spleens, scale bars represent 500 μm (**j**), and quantified with automated hCD20+ IHC analysis (**k**); box plots indicate median (middle line), 25th, 75th percentile (box) and minimum and maximum (whiskers) and the statistical analysis was performed by Mann–Whitney test. Automated detection of hCD20 is depicted in Supplementary Fig. S3e. Significance values are depicted in the graph; (n.s.) not significant. Source data are provided as a Source Data file.

To test whether transient inactivation of SHIP1 is a potential therapeutic option for CLL, we assessed disease progression in different in vivo models of CLL. To determine the effects of SHIP1 inhibition on CLL in an immunocompetent system, we again used the TCL1tg CLL mouse model^33^, which is an established tool for studying therapeutic targets in human CLL^47^. Here, we evaluated the disease progression in the peripheral blood upon transplantation of murine donor CLL cells (mCLL) that revealed a relatively indolent progression in vivo and initiated the 3AC treatment when the CLL fraction was clearly detectable in the peripheral blood (d8, Fig. 3a). The treatment schedule is shown in Supplementary Fig. 3a. We then followed the expansion of CLL cells in the peripheral blood over time and observed a significant reduction in the CLL progression in the SHIP1 inhibitor-treated mice (Fig. 3b). We then analyzed the CLL infiltration in the secondary lymphoid organs 30 days post CLL injection. All CLL target organs revealed significantly less CLL cell infiltration in the 3AC-treated group as compared to the control group (Fig. 3c). To also mimic therapeutic approaches in CLL patients that already have high levels of CLL in the peripheral blood at the time of treatment initiation, we also tested the efficacy of SHIP1 inhibition in a highly aggressive mCLL where the disease had progressed to ~50% CD19^+^ CD5^+^ cells in the peripheral blood at the time of treatment initiation (Fig. 3d). Similar to our previous results, treatment with 3AC diminished the progression of the disease in the peripheral blood (Fig. 3e) and reduced the levels of remaining CLL in all target organs of murine CLL, despite the short treatment schedule of 8 doses (Fig. 3f, treatment schedule is shown in Supplementary Fig. S3b). To also assess the efficacy of SHIP1 inhibition on human CLL in vivo, we treated NOD/SCIDcγ^−/−^ (NSG) mice xenografted with primary patient-derived CLL cells with the SHIP1-specific inhibitor 3AC in vivo (treatment schedule shown in Supplementary Fig. 3c). The gating strategy for the analysis of primary CLL cells is depicted in Supplementary Fig. 3d. Equal engraftment of hCLL cells was confirmed prior to injection (Fig. 3g). Similar to the treatment of murine CLL, we observed a significant reduction in the amount of hCLL cells in CLL target organs in the 3AC treatment group as compared to the control group, determined by flow cytometric analysis (Fig. 3h, i) and histology (using immunohistochemistry detecting human CD20 in the spleen, Fig. 3j, k). The HE staining of the histology with hCD20 analysis and labeling for statistical evaluation of sections derived from five mice per group is shown in Supplementary Fig. 3e. The SHIP1-specific inhibitor 3AC did not induce toxicity in vivo as indicated by the constant body weight of the treated mice pre- and post-treament (Supplementary Fig. 3f). Together, these results reveal that transient pharmacological SHIP1 inhibition represents a potential therapeutic strategy for treating murine and human CLL in vivo.

### Genetic validation of effects mediated by SHIP1 inhibition

**Fig. 4:**
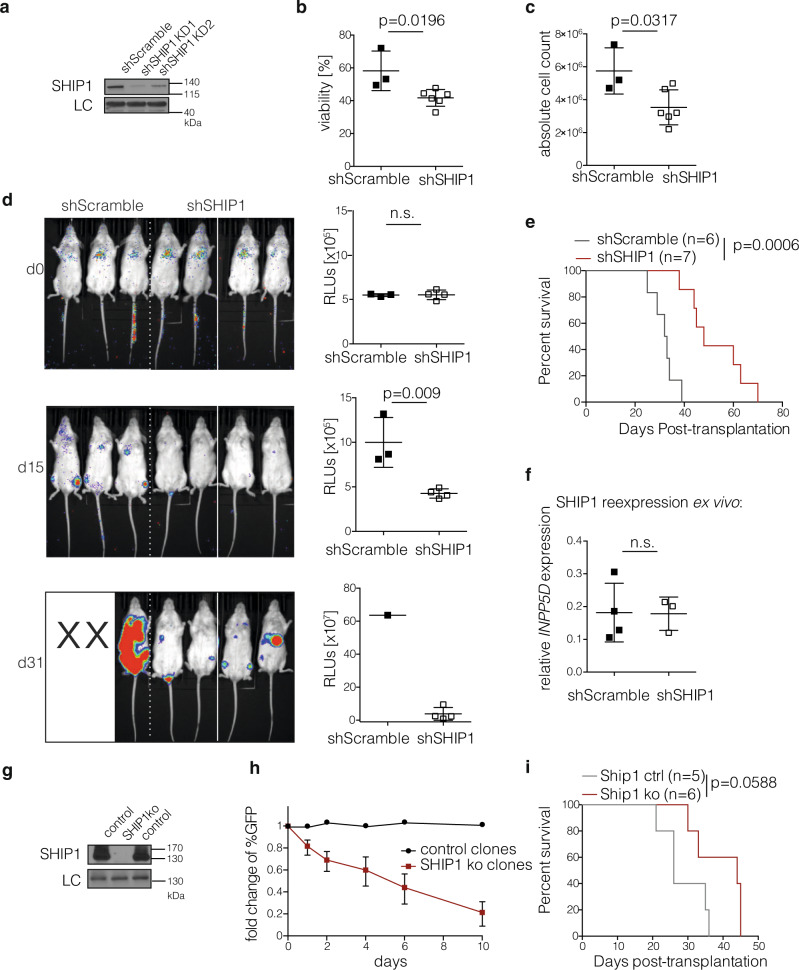
Genetic validation of SHIP1 inhibitor effects. **a** Immunoblot analysis of SHIP1 in MEC-1 cells stably transduced with two different SHIP1 shRNA knockdown constructs (KD1 and KD2). β actin serves as a loading control (LC), representative for two independent experiments. **b** Viability of MEC-1 cells upon transduction and selection with SHIP1 shRNA (shSHIP1; clear squares) or scrambled constructs (shScramble; black squares) was determined via flow cytometry and DAPI exclusion, summary of three independent experiments is shown, shSHIP1 pooled data using KD1 and KD2 targeting vectors. Data are presented as individual values and mean values ± SD. Statistical significance was assessed by a two-tailed unpaired Student’s *t*-test. **c** Absolute number of MEC-1 cells upon transduction and selection with shScramble (black squares) and shSHIP1 (clear squares; pooled data using KD1 and KD2) constructs, summary of three independent experiments is shown. Data are presented as individual values and mean values ± SD. Statistical significance was assessed by a two-tailed unpaired Student’s *t*-test. **d** Bioimaging upon injection of MEC-1 Eco/Luc transduced with SHIP1 shRNA (KD1 or KD2; *n* = 4) or scrambled shRNA (*n* = 3) containing cells into NSG mice. 1 h after injection, equal loading with MEC-1 cells was confirmed. The progression was monitored on day 15 and day 31, as indicated, quantification of the light signal was performed using the Living Image Software. X indicates mice that had already succumbed to the disease. Data for quantification are presented as individual values and mean values ± SD. Statistical significance was assessed by a two-tailed unpaired Student’s *t*-test. **e** Kaplan–Meier Survival analysis is shown for NSG mice injected with MEC-1 carrying SHIP1 shRNA (red line; *n* = 7; KD1 and KD2) or shScramble (gray line; *n* = 6) constructs, pooled analysis of two independent experiments. Statistical significance between the survival curves with the corresponding *p*-value was calculated by a log-rank (Mantel–Cox) test. **f** MEC-1 cells with control or SHIP1 shRNA were isolated from diseased mice (*n* = 4 and *n* = 3, respectively) and analyzed for SHIP1 expression via qPCR and normalized to the housekeeping gene GAPDH. Data are presented as individual values and mean values ± SD. Statistical significance was assessed by a two-tailed unpaired Student’s *t*-test. **g** Western blot analysis of single-cell clones transduced with *INPP5D*-targeting guide RNAs and Crispr/Cas9. One successful SHIP1ko clone is shown, DHX9 serves as a loading control (LC). Representative analysis for at least four independent clones. **h** Growth competition assay of four successfully generated, independent GFP^+^ SHIP1 knockout clones (red squares) and 2 GFP^+^ control clones (black circles) by mixing with the GFP-negative parental MEC-1 cells. Data are presented as mean values (with ±SD for knockout clones) of the fold change of GFP expression over time. **i** SHIP1 wt (gray line; *n* = 4) and knockout MEC-1 cells (red line; *n* = 5) were injected into NSG mice and symptom-free survival was assessed. Statistical significance was assessed by log-rank (Mantel–Cox) test. Significance values are depicted in the graph; (n.s.) not significant. Source data are provided as a Source Data file.

In order to validate on-target effects of SHIP1 inhibitors mediating the observed cytotoxic effects, we employed genetic loss-of-function studies. We first performed shRNA knockdown experiments using the CLL-derived cell line MEC-1. We confirmed reduced SHIP1 protein levels with two *INPP5D*-targeting vectors (shSHIP1 KD1 and KD2) as compared to scrambled shRNA control cells with immunoblot (Fig. 4a). When analyzing the viability of MEC-1 cells with SHIP1 knockdown, we observed a reduction in viability (Fig. 4b) and cell count upon SHIP1 knockdown (Fig. 4c), similar to the effects observed upon 3AC treatment. To investigate the relevance of SHIP1 expression in vivo, we transplanted MEC-1 cells carrying scrambled control shRNA or SHIP1-targeting shRNA into NSG mice, a model for aggressive, rapidly progressing human CLL^48^. Based on the different viability rates at the time of cell injection, we used luciferase-expressing MEC-1 cells that can be detected via bioimaging in vivo. We conducted bioimaging on the day of cell injection to confirm similar amounts of viable MEC-1 cells in all transplanted mice (Fig. 4d, top panel). However, over a period of 4 weeks, SHIP1 knockdown significantly reduced the MEC-1 expansion in vivo as indicated by a reduced light signal detected via bioimaging (Fig. 4d, lower panels). MEC-1 SHIP1 knockdown-bearing mice lived significantly longer than those that received MEC-1 cells with scrambled shRNA vectors (Fig. 4d, e). Importantly, reanalysis of SHIP1-targeting shRNA carrying MEC-1 cells obtained from mice with active disease revealed that these expressed SHIP1 at levels similar to those in the control MEC-1 cells, indicating that the clones that escaped the knockdown outgrew and formed lethal leukemia (Fig. 4f). In addition, we performed CRISPR/Cas9-mediated knockout of the *INPP5D* gene in MEC-1 cells. After successful validation of targeting through western blot (Fig. 4g) and gene sequencing (Supplementary Fig. 3g), we performed competitor growth assays and confirmed the selective disadvantage of SHIP1-deficient MEC-1 cells in vitro (Fig. 4h) and a similar trend was observed in vivo (Fig. 4i). Note that SHIP1 knockout MEC-1 cells were expanded from single cells for several weeks prior to these experiments and may have adapted to higher PI3K/AKT signaling levels during this period. This adaptation could weaken the growth-limiting effects of SHIP1 deletion as compared to the acute inhibitor treatments. To further assess the specificity of our SHIP1 inhibitor, we confirmed that SHIP1-deficient MEC-1 cells were significantly less sensitive to 3AC treatment as compared to control clones (Supplementary Fig. 3h), confirming that the cytotoxic effect is largely due to on-target SHIP1 inhibition. Together, our data clearly shows that SHIP1 expression and activity is required for optimal CLL cell growth and survival.

### PI3K/AKT hyperactivation promotes oxidative phosphorylation in CLL cells followed by ROS-mediated cell death

**Fig. 5:**
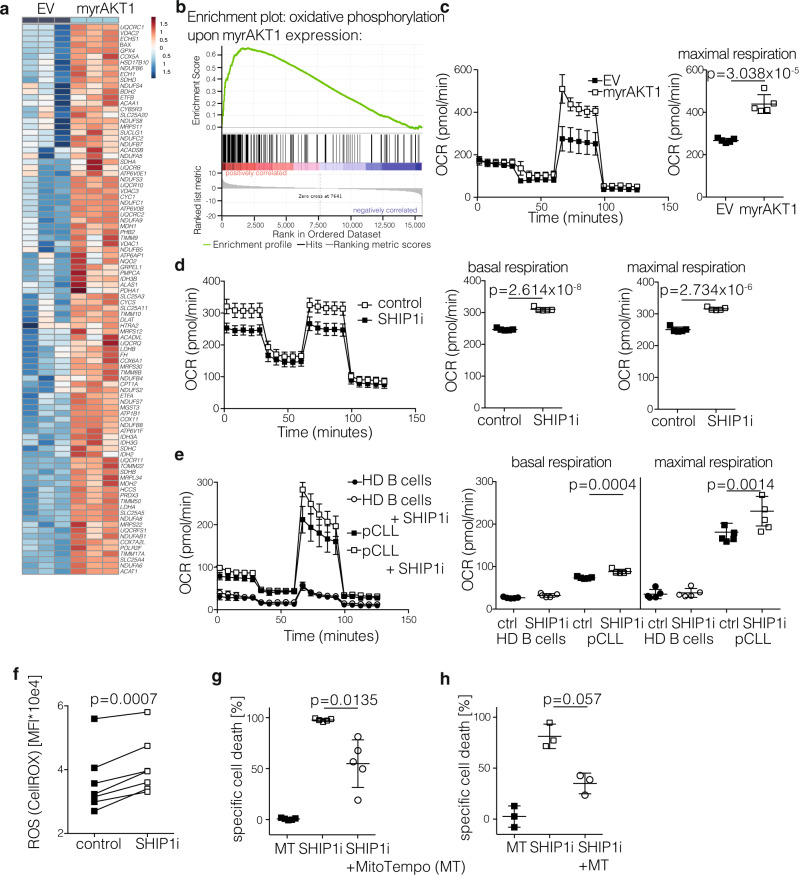
Mechanistic analysis of AKT1 activation/SHIP1 inhibition in CLL. **a** Heatmap analysis of differentially regulated genes associated with oxidative phosphorylation in MEC-1 cells upon transduction with pMIG EV (left) or pMIG-myrAKT1 (right), representative for two independent experiments. Color scale indicates the *Z* score (red = up; blue = down). **b** Gene set enrichment analysis (GSEA) results for the association with “oxidative phosphorylation” is shown upon myrAKT expression, Enrichment Score (ES) 0.65666306; Normalized Enrichment Score (NES) 2.847461; Nominal *p*-value < 0.001; FDR *q*-value < 0.001; FWER p-value < 0.001; representative for two independent experiments. **c** Oxygen consumption rate (OCR) was measured in GFP-sorted MEC-1 cells upon transduction with pMIG EV or pMIG-myrAKT1, pooled analysis of three independent experiments. Data are presented as mean values ± SEM (left) and as individual values and mean values ± SD in the bar graph (right). Statistical significance was assessed by a two-tailed unpaired Student’s *t*-test. **d** OCR was measured in MEC-1 cells treated for 1 h with SHIP1 inhibitor (SHIP1i; 5 μM 3AC; clear squares) or control (black squares), pooled analysis of seven independent experiments. Data are presented as mean values ± SEM over time (left) and as individual values and mean values ± SD in the bar graph (right). Right: Statistical significance was assessed using mean values of three independent experiments by a two-tailed unpaired Student’s *t*-test. **e** OCR was measured in a healthy donor (HD) B cells (*n* = 6; circles) and primary CLL cells (*n* = 6, squares) upon 1 h treatment with SHIP1i (5 μM 3AC; clear) or control (filled); pooled data from two independent experiments. Data are presented as mean values ± SEM (left) and as individual values and mean values ± SD in the bar graph (right). Statistical significance was assessed by a two-tailed unpaired Student’s *t*-test. **f** Quantification of MFI upon ROS analysis using the CellROX orange dye in primary CLL cells untreated, or treated for 4 h with 5 μM 3AC is shown (*n* = 8). Data are presented as individual values per CLL donor. Statistical significance was assessed by a two-tailed paired Student’s *t*-test. **g** MEC-1 cells were treated with MitoTEMPO (MT; filled squares), SHIP1i (3AC; clear squares) or the combination (clear circles) for 24 h treatment, measured in five independent experiments; the specific cell death was determined as described in Fig. 2e. Data are presented as individual values and mean values ± SD. Statistical significance was assessed by a two-tailed unpaired Student’s *t*-test. **h** Primary CLL samples (*n* = 3) were treated with MT (filled squares), SHIP1i (3AC; clear squares), or the combination (clear circles) for 24 h; the specific cell death was determined as described in Fig. 2e. Data are presented as individual values and mean values ± SD. Statistical significance was assessed by a two-tailed unpaired Student’s *t*-test. Significance values are depicted in the graph; (n.s.) not significant. Source data are provided as a Source Data file.

To elucidate the mechanism of how hyperactivation impairs CLL progression, we again employed the genetic strategy of PI3K/AKT hyperactivation by forced constitutively active AKT1 expression in MEC-1 cells. After sorting of GFP^+^ myrAKT1 or EV-expressing MEC-1 cells, we conducted RNAseq analysis with subsequent gene set enrichment analysis^49^ and found a significant upregulation of genes related to “oxidative phosphorylation” upon AKT1 activation in MEC-1 cells (Fig. 5a, b and Supplementary Fig. 4a, Supplementary Data 1). Similar effects were observed in MEC-1 cells upon shRNA-mediated SHIP1 knockdown (Supplementary Fig. 4b). This upregulation was rather surprising, as PI3K/AKT signaling is primarily known to promote the glycolytic metabolic pathway to ensure fast responses to energy demands^50^. To test the metabolic status functionally, we measured the metabolic consequences of AKT1 activation as well as SHIP1 inhibition in CLL cells. Without manipulation, CLL cells primarily rely on mitochondrial oxidative phosphorylation for energy supply^51^. Accordingly, we analyzed mitochondrial functions by determining the oxygen consumption rate (OCR) in MEC-1 cells overexpressing myrAKT1 or empty vector control cells and found higher mitochondrial respiration capacity in the myrAKT1-expressing CLLs (Fig. 5c), which is in line with the enriched “oxidative phosphorylation” gene expression signature. Similarly, treatment of MEC-1 cells with the SHIP1 inhibitor 3AC increased OCR levels, both at the baseline and upon respiratory challenge (Fig. 5d). Finally, primary CLL cells also revealed increased mitochondrial capacity upon SHIP1 inhibition, which was less pronounced in healthy donor B cells (Fig. 5e). Glycolytic activity measured by the extracellular acidification rate (ECAR) was not significantly affected by 3AC treatment (Supplementary Fig. 4c). During oxidative phosphorylation, electrons escape from the electron transport chain to induce formation of reactive oxygen species (ROS), including superoxide anions and hydrogen peroxide^52^. To investigate whether the increase in mitochondrial respiration upon SHIP1 inhibition impacts ROS levels in CLL, we measured levels of cellular ROS upon SHIP1 inhibition and indeed observed that the high levels of ROS in primary CLL cells^51^ were further increased by 3AC treatment (Fig. 5f). Similarly, in the CLL-derived cell line MEC-1 we found upregulated ROS levels in SHIP1 knockout clones as compared to controls of the MEC-1 line. Treatment of SHIP1 knockout clones with 3AC, however, did not further increase ROS levels (Supplementary Fig. 4d–g), confirming that 3AC-mediated ROS induction is due to on-target SHIP1 inhibition. Importantly, blocking ROS levels derived from oxidative phosphorylation with a mitochondria-targeted antioxidant (mitoTEMPO) significantly reduced the cytotoxicity of SHIP1 inhibition in MEC-1 cells (Fig. 5g) and primary CLL cells (Fig. 5h). Similar results were obtained with the ROS scavenger N-acetyl-cyteine (NAC; Supplementary Fig. 4h, i). These results indicate that PI3K/AKT pathway activation in CLL (by myrAKT1 overexpression, genetic SHIP1 deletion, or by SHIP1 inhibition) promotes the oxidative metabolic pathway in CLL and thereby leads to the formation of toxic ROS levels.

### SHIP1 inhibition induces lytic cell death with immunogenic features

**Fig. 6:**
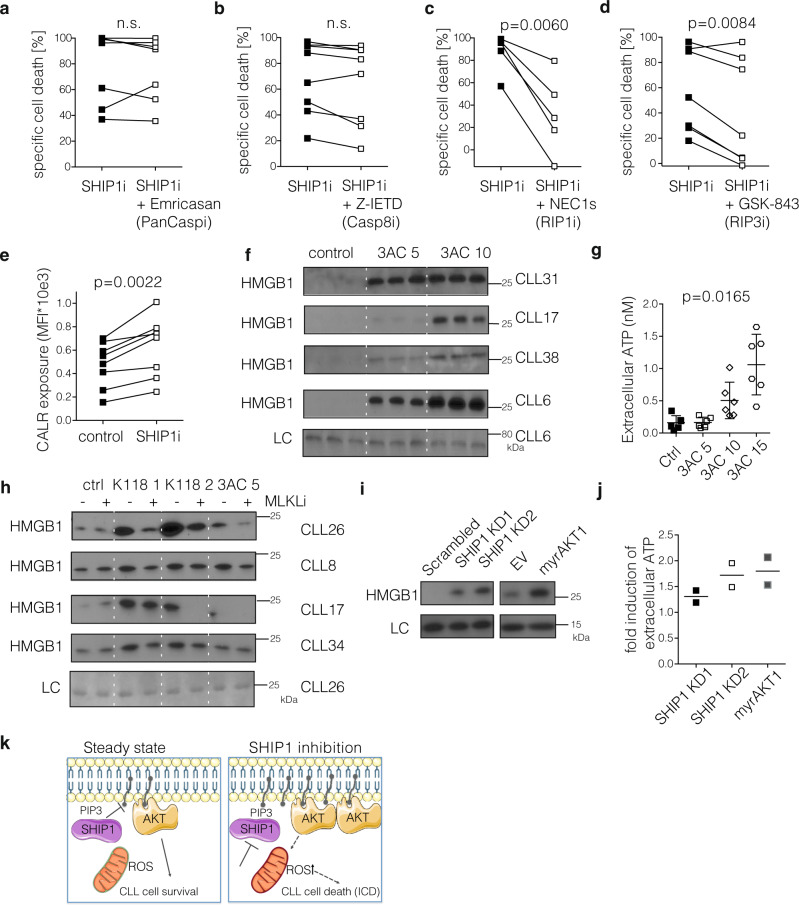
SHIP1 inhibition induces lytic cell death with immunogenic features. **a**–**d** Primary CLL samples were treated with the SHIP1 inhibitor (SHIP1i) 3AC or the combination of 3AC with the Caspase inhibitor (PanCaspi) Emricasan (**a** *n* = 7), the Caspase-8 inhibitor (Casp8i) Z-IETD (**b** *n* = 8), the RIP1 inhibitor (RIP1i) NEC1s (**c** *n* = 5) or the RIP3 inhibitor (RIP3i) GSK-843 (**d** *n* = 7) and the specific cell death was determined as described in Fig. 2e. For the combination treatment, the viability of the single treatments of the cell death inhibitors was used for determining the baseline cell death to calculate the specific cell death. Viability is depicted in Supplementary Fig. 4j–m; Data are presented as individual values and statistical significance was assessed by a two-tailed paired Student’s *t*-test. **e** Calreticulin (CALR) exposure to the outer membrane is determined after 4 h of 3AC treatment on primary CLL samples (*n* = 8) as determined by flow cytometric analysis. Data are presented as individual values and statistical significance was assessed by a two-tailed paired Student’s *t*-test. **f** Supernatant of four primary CLL samples was collected after 48 h and HMGB1 levels determined by immunoblot. A representative loading control (unspecific band at 15 kDa) is shown for one CLL sample. **g** Dose-dependent accumulation of extracellular ATP levels in the supernatants of primary CLL samples (*n* = 6) was determined after 4 h of treatment with the SHIP1 inhibitor 3AC. Data are presented as individual values and mean values ± SD. Statistical significance was assessed by an ordinary one-way ANOVA test. **h** Supernatant of four primary CLL samples was collected after 48 h with the SHIP1/2 inhibitor K118 1, 2, and 3AC 5 μM and in combination with the MLKL inhibitor (MLKLi) NSA (2 μM), and HMGB1 levels determined by immunoblot. A representative loading control is shown for one CLL sample. **i** Supernatants of puromycin-selected (shRNA expressing, d6) or GFP-sorted (EV, myrAKT expressing, d5) MEC-1 cells were analyzed for HMGB1 content. An unspecific band appearing at 15 kD served as a loading control. Representative example for two independent experiments. **j** Fold change to control of extracellular ATP levels in the supernatants of MEC-1 cells after SHIP1 knockdown (shSHIP1 KD1 (black squares) and KD2 (clear squares) vs. shScrambled, d6 post selection) and after myrAKT overexpression (gray squares; myrAKT vs. pMIG, d5 post sorting), individual values and mean value of two independent experiments are shown. **k** Cellular consequences of transient SHIP1 inhibition in CLL are illustrated (top panel) as compared to steady-state status (bottom panel), this figure was created using Servier Medical Art templates (https://smart.servier.com). Source data are provided as a Source Data file.

Given that ROS accumulation plays a key role in mediating different forms of programmed cell death^53,54^, we next investigated which mode of cell death is induced in CLL upon SHIP1 inhibition and tested different inhibitors of cell death pathways in combination with 3AC treatment of primary CLL samples. While pan-caspase- or caspase-8 inhibition had very little or no effect on the cytotoxicity induced by 3AC (Fig. 6a, b), necroptosis inhibition by NEC1s (inhibiting RIP1) or GSK-843 (inhibiting RIP3) did significantly reduce the 3AC-mediated cytotoxicity in primary CLL (Fig. 6c, d). The respective viabilities after the indicated treatments (alone and in combination) are depicted in Supplementary Fig. 4j–m. As necroptosis is an immunogenic form of cell death, we further evaluated whether CLL cells acquired characteristics associated with immunogenic cell death (ICD)^55^ upon treatment with the SHIP1 inhibitor 3AC. Translocation of calreticulin to the surface of stressed cells acts as “eat me” signal for their removal by phagocytosis^56^. As we expected, calreticulin was exposed to the outer membrane in CLL cells upon SHIP1 inhibition (Fig. 6e). In addition, CLL cells secreted the danger-associated molecular pattern (DAMP) HMGB1 (Fig. 6f) and ATP to the supernatants upon 3AC treatment in a dose-dependent manner (Fig. 6g), which also contributes to the activation of adjacent immune cells. Importantly, in the untreated control CLL cells, no HMGB1 and only low levels of extracellular ATP were detected, despite the high level of spontaneous apoptotic cell death after 48 h in primary CLL cell cultures. This indicates that the release of these immunostimulatory molecules by CLL cells is specifically induced by SHIP1 inhibition rather than secondary, unspecific events during spontaneous cell death. In line with this assumption, HMGB1 release upon SHIP1 inhibition can be partially blocked by inhibiting the MLKL-mediated pore formation using small-molecule inhibition (Fig. 6h). We also observed increased levels of HMGB1 and ATP secreted upon shRNA-mediated SHIP1 knockdown and after myrAKT expression in MEC-1 cells (Fig. 6i, j). These results indicate that SHIP1 inhibition induces a lytic form of cell death with features of necroptosis that triggers the release of immunogenic mediators from CLL cells in vitro. Taken together, we, therefore, conclude that SHIP1 inhibition induces a lytic form of cell death with immunogenic features (summarized in Fig. 6k).

## Discussion

In this study, we identified a previously unrecognized vulnerability of CLL cells to acute and constitutive activation of the PI3K/AKT signaling pathway. We confirmed that the expression and activity of the inhibitory phosphatase SHIP1 is required to limit PI3K/AKT signaling in CLL cells to prevent excessive ROS production and thereby avoid an immunogenic form of cell death. We, therefore, suggest transient pharmacological targeting of SHIP1 as a therapeutic approach for CLL.

When we initiated our investigation of what renders CLL cells specifically vulnerable to AKT1 activation or SHIP1 inhibition, we found that additional activation of AKT1 increases oxidative phosphorylation, thereby triggering ROS-dependent cell death in CLL. In many solid tumors, malignant cells rely on the fast but inefficient generation of ATP via glycolysis to maintain their energy demands in a hypoxic condition with dysfunctional mitochondria. AKT activation has emerged as a central player in this metabolic switch. However, similar to non-transformed, activated B cells, CLL cells circulate in normoxic conditions and primarily rely on mitochondrial oxidative phosphorylation for energy supply, despite their active AKT1 signaling^51,57,58^. Therefore, CLL cells already have high levels of ROS and have evolved mechanisms to cope with this level of oxidative stress, i.e., via upregulation of the antioxidant enzyme hemoxidase 1^51^. Enforced increase of AKT signaling, either via SHIP1 inhibition or genetic activation, however, leads to a further increase in oxidative phosphorylation and to ROS levels toxic for CLL cells. It should be noted that SHIP1 can also have activating roles in cell signaling as PI(3,4)P_2_, the product of SHIP1 dephosphorylating PI(3,4,5)P_3_, can also result in AKT activation^59^. However, our data clearly shows that SHIP1 inhibition results in CLL cell activation, and this activation is critical for SHIP1 inhibition-mediated cytotoxicity. In addition, although genetic PI3K/AKT signaling activation upon introduction of constitutively active mutant AKT1 induces stronger and more persistent signaling compared to SHIP1 inhibition or deletion, we still observed surprisingly similar downstream effects. These effects include the induction of oxidative phosphorylation and ROS accumulation followed by immunogenic cell death. Nevertheless, the induction of cell death was weakened in the genetically manipulated MEC-1 cells as compared to the inhibitor-treated cells, and we speculate that this weakened effect is largely due to adaptation to the higher signaling levels as genetic manipulation takes days and even weeks to successfully create SHIP1 knockdown/knockout lines. In line with the assumption of cellular adaption, genetic knockout of SHIP1 in B cells does not delay the disease development in the TCL1tg mouse model where pre-malignant cells can develop strategies to counteract hypersignaling prior to full transformation^60^.

AKT activation has also been linked to promoting cell death in non-hematopoietic cells, particularly if the cells are metabolically challenged^61,62^. Our study demonstrates that AKT activation and increased levels of ROS in CLL cells are critical downstream mediators of SHIP1 inhibition in CLL resulting in a lytic form of cell death. In line with our finding, the induction of necroptotic cell death by TNFα and Caspase inhibition (Z-VAD) in neurons is preceded by the assembly of the critical necroptosis kinases RIP1-RIP3 with activated AKT, and, similar to our observation, pretreatment with small molecule inhibitors of AKT prevented the formation of ROS and necroptosis^63^. Increased ROS levels can be directly detected by RIP1, leading to its activation by autophosphorylation on serine residue 161^64^. This specific phosphorylation then recruits RIP3 and induces the formation of a functional necrosome with pore formation and the release of DAMPs^65^. We observed that RIP1 and RIP3 kinase activity is critical for SHIP1 inhibition-induced cytotoxicity in CLL and this is particularly interesting as we observe features of immunogenic cell death. We further confirmed that CLL cells exhibit key characteristics of immunogenic cell death with the release of immunostimulatory molecules upon SHIP1 inhibition. We hence provide evidence that SHIP1 inhibition promotes an immunogenic form of lytic cell death in CLL, which could be contributing to the treatment efficacy.

We had initiated our study based on the hypothesis that strong activation of the PI3K/AKT pathway mimics excessive signaling strength from an autoreactive BCR and that this may be leveraged to trigger negative selection for therapeutic benefit in CLL patients. It is unlikely that autoreactive B cells undergo an immunogenic form of cell death during physiological B-cell selection but most likely undergo classical apoptosis. In T cells, SHIP1 inhibition induces classical apoptosis via Fas/Caspase-8^66^, while our experiments indicate that 3AC-mediated cell death in CLL is largely independent of caspases. One possible explanation for the differential mode of cell death triggered by SHIP1 inhibition in CLL is the upregulated antiapoptotic machinery in CLL, one of the hallmarks of this disease: CLL cells express high ratios of c-FLIP(L) to caspase-8, and thereby prevent caspase-8 activation and apoptosis^67^, which can promote the induction of a necroptotic form of cell death^68^. The sensitivity of CLL cells towards excessive signaling may therefore still resemble negative B-cell selection while the induction of an immunogenic form of cell death upon SHIP1 inhibition specifically occurs in transformed CLL cells.

Nevertheless, targeting negative regulators such as SHIP1 also risks promoting unwanted proliferation of other, non- or pre-malignant cells. Indeed, B-cell-specific knockout of SHIP1 in mice results in the loss of B-cell tolerance and autoimmune manifestations^69^. Similarly, SHIP1 may act as a tumor suppressor in other tumor entities, including AML and T-ALL, and its inhibition may promote proliferation of pre-malignant cells of other origins^70^. However, our therapeutic approach is based on transient SHIP1 inhibition, and potential proliferative signals to other cells are only present for the limited time of treatment. In our short-term treatment schedule in mice, we did not observe any evidence of side effects from SHIP1 inhibition, neither in *wt* nor in NSG mice. Similarly, other groups reported extended survival of mice challenged with multiple myeloma^71^ with no apparent toxicity upon SHIP1 inhibitor in vivo treatment, even upon continuous treatment with 3AC in immunocompetent mice^44,72^.

We hypothesize that some of the effects of SHIP1 inhibition on other, non-CLL cells may also increase treatment efficacy. Recent studies have demonstrated that SHIP1 inhibition in different tumor models can increase the anti-tumor NK- and T-cell responses^73^. This is particularly promising in the context of CLL where patients suffer from drastic immunosuppression leading to fatal infections causing up to 30–50% of CLL-related mortalities^74^. We, therefore, suggest that both cell autonomous and nonautonomous mechanisms can contribute to the therapeutic efficacy of SHIP1 inhibition in CLL. We speculate that transient SHIP1 inhibition in repetitive cycles can cause lytic cell death with immunogenic features in the malignant cells and simultaneously enhance the immunoresponse by directly acting on NK- and T cells to restore effective immune responses and potential anti-tumor immunity in CLL.

Taken together, our results show that CLL cells rely on a delicate coordination between the cellular signaling pathways regulating metabolic processes for their cellular growth and depend on intermediate signaling. We show that perturbation of negative regulation of the PI3K/AKT signaling, even if counter-intuitive, induces a ROS-mediated lytic and immunogenic form of cell death in CLL. We, therefore, propose transient inhibition of SHIP1 as an unexpected concept for CLL therapy, either alternating with kinase inhibition to potentially enhance the effect of both solo treatments or to treat the rising cases of kinase inhibitor-resistant disease.

## Methods

### Human subjects

Primary CLL samples were obtained from the peripheral blood of patients at the National Center for Tumor Diseases, Heidelberg, Germany (CLL1-CLL30, Supplementary Table 1). Data for IgV_H_ status, SHIP1 expression was obtained by RNA Sequencing^39^. Clinical data and gene expression were analyzed by Junyan Lu (EMBL Heidelberg). In addition, Klinikum München Schwabing (Clemens Wendtner; CLL31-CLL36) and Tumor Therapy Center of Klinikum rechts der Isar (Christian Bogner, CLL37-CLL44) provided peripheral blood of CLL patients for xenotransplantation approaches and in vitro experiments. The local ethics committee of the Faculty of Medicine, Technical University Munich, approved patient and healthy donor sampling and all presented experiments. All participants (CLL patients and healthy donors) gave informed consent. All patients were treatment naïve or off CLL therapy for at least 3 months. Healthy donor-derived blood samples (age-matched) were received from the “Bayerisches rotes Kreuz” (Munich, Germany).

### Cell lines

Chronic B-cell leukemia-derived cell lines MEC-1 (RRID: CVCL_1870), EHEB (RRID: CVCL_1194), and lymphoma lines SUDHL6 (RRID: CVCL_2206), BJAB (RRID: CVCL_5711), and Bal17 (RRID: CVCL_9474) were purchased from DSMZ (Braunschweig, Germany). MEC-1 (Slc7a) Eco cells were generated by transduction with pLenti6/UbC/mSlc7a1, a gift from Shinya Yamanaka (Addgene plasmid # 17224; RRID: Addgene_17224) and MEC-1 Luciferase positive cells were generated by the introduction of pCL6-Luc-GFP into MEC-1 Eco cells by retroviral transduction.

### Quantitative real-time PCR (qPCR)

RNA was isolated from sorted bone marrow- or spinal cord-residing MEC-1 cells by using RNeasy Plus Micro Kit (QIAGEN) according to the manufacturer’s instructions. RNA concentration of the samples was determined by NanoDrop. RNA was reverse transcribed using the qScript cDNA SuperMix (Quantabio) according to the manufacturer’s instructions with 20–1000 ng of total RNA. The generated cDNA was used in triplicates for RT-PCR reactions, with primers that span exon–exon boundaries to ensure cDNA-specific amplification. The qPCR Kit Takyon™ No ROX SYBR® 2X MasterMix dTTP Blue (Eurogentec) was used to perform RT-PCR. Gene expression was normalized to the housekeeping gene GAPDH. The reaction was performed in a Light Cycler 480 II (Roche). All primer sequences are listed in Supplementary Table 2.

### myrAKT1 in MEC-1

pMIG-emtpy vector (EV) and pMIG-myrAKT1 were kindly provided by Hassan Jumaa^32^ and by retroviral transduction introduced into MEC-1 Eco cells. GFP content was followed over time by flow cytometric analysis and DAPI exclusion. The expression of active AKT was confirmed by western blot.

### Mice

Mice were housed according to the guidelines specified in the EU Directive 2010/63 with a light-dark rhythm of 12 h each with twilight phase, air condition (at 20–24 °C temperature), and a humidity of 45–60%. The AKT1^E17K^ cDNA, carrying a point mutation at nucleotide position 49 (G to A) in the human AKT1 gene, was cloned into the ubiquitously expressed ROSA26 vector, preceded by a loxP-flanked transcriptional and translational STOP cassette. Electroporation of 129J/Ola embryonic stem cells and generation of chimeric mice were performed by Polygene, Switzerland. Successful recombination of embryonic stem cell clones was evaluated by Southern Blot analysis of genomic DNA digested with *Xba*I^36^. Germline transmission was confirmed by PCRs specific for the targeted locus. All primers used are listed in Supplementary Table 2. The bicistronic expression of AKT1^E17K^ together with eGFP preceded by an internal ribosomal entry site (IRES) sequence allowed fluorescence monitoring of AKT1^E17K^ expressing cells. Blastocyst injection of the clones and subsequent chimera breeding resulted in AKT1^E17K^ transgenic mice that were then crossed to Mb1-CreER^T2^ mice^37^ and the TCL1tg mouse model^33^.

For xenotransplant experiments, we used NSG mice (*NOD.Cg-Prkdcscid Il2rgtm1WjI/SzJ*), purchased from Charles River or Janvier laboratory as recipients for the human MEC-1 CLL-like cell line (purchased from DSMZ, Braunschweig, Germany) or primary CLL patient samples. For treatment of murine CLL in vivo, we transplanted 2 × 10e7 splenocytes of aged TCL1tg mice^33^ into C57BL/6N (Janvier Labs) *wt* immunocompetent mice and waited for detection of CLL or full engraftment and disease progression. Treatment schedules are depicted in Supplementary Fig. 3. All animal experiments were carried out in accordance with the guidelines of the Federation of European Laboratory Animal Science Association (FELASA) and followed the legal approval of the Government of Upper Bavaria (Regierung von Oberbayern).

### AKT1 hyperactivation in the TCL1tg model via Cre-mediated recombination in vitro and in vivo

Isolated splenocytes of AKT1^E17K^ × TCL1tg or TCL1wt littermates were treated with 500 ng/ml 4-OHT for 48 h or with TAT-Cre (Excellgen) diluted in Opti-MEM at a final concentration of 2 μM for 1 h at 37 °C, 5% CO_2_, and 95% humidity. Afterward, cells were washed twice in culture media. The induction of the AKT1 transgene was analyzed by flow cytometric measurement of GFP percentage and DAPI exclusion. To activate AKT1^E17K^ in vivo, mice transplanted with above described leukemic triple transgenic splenocytes were induced to express AKT1^E17K^ by providing tamoxifen-enriched chow (400 mg tamoxifen citrate kg^–1^ chow; CreActive TAM400, LASvendi, Soest, Germany) or control chow for 4 weeks upon transplantation to activate CreER^T2^. CLL content and GFP expression in the peripheral blood were followed by flow cytometry and after 2-month mice were sacrificed, organs harvested, and analyzed by flow cytometry.

### Cell culture

The murine B-cell lymphoma cell line BAL17 and freshly isolated primary murine B and T cells were cultured in RPMI-1640 Glutamax medium supplemented with 10% FBS, 1% PenStrep, and 0.1% 2-mercaptoethanol. Patient-derived CLL and healthy donor-derived B cells, as well as EHEB, BJAB, and SUDHL6 cell lines were cultured in RPMI-1640 Glutamax medium supplemented with 10% FBS and 1% PenStrep. CLL cell line MEC-1 was cultivated in IMDM medium supplemented with 10% FBS and 1% PenStrep. HEK293T cells for virus production were grown in DMEM supplemented with 10% FBS and 1% PenStrep. All cells were cultured under standard cell culture conditions; at 37 °C, 5% CO_2_, and 95% humidity.

### Cell isolations

Peripheral blood mononuclear cells (PBMCs) were isolated from whole blood by density gradient centrifugation using Ficoll-Paque (GE Healthcare, Chicago, IL, USA). CD19^+^ B cells, including MEC-1 cells, were purified by magnetic-activated cell sorting (MACS) using human B-cell isolation kit II or human CD19 MicroBeads (Miltenyi, Bergisch-Gladbach, Germany). Purification of primary CLL cells (CD19^+^ CD5^+^) and MEC-1 cells (GFP, CD19^+^) was performed using fluorescence-activated cell sorting (FACS) (BD Aria II, BD Bioscience, Franklin Lakes, NJ, USA). All antibodies are listed in Supplementary Table 3. Peripheral blood, spleen, and axillary lymph nodes were harvested per mouse. Organs were meshed through a 70 μm cell strainer in PBS buffer and erythrocytes were lysed using G-DEXTMIIb RBC Lysis Buffer (Intron Biotechnologies).

### Western blot

Whole-cell lysates for protein analysis were prepared in CHAPS buffer, supplemented with phosphatase inhibitors (50 mM NaF, 0.1 mM Na_3_VO_4_, and protease inhibitors in resolved EDTA-free Protease Inhibitor Cocktail Tablets, Roche Diagnostics) for 15 min on ice using standard methods. BCA Protein Assay Kit (ThermoFisher Scientific) was applied for protein concentration determination. 4–12% gradient gels from Invitrogen, NuPage were applied according to manufacturer’s instructions. To analyze the release of HMGB1, supernatants were collected upon 48 h 3AC treatment by centrifugation (400 × *g*, 5 min, 4 °C) and Laemmli buffer was added, according to standard protocols. Samples were loaded on 10% Tris/Bis gels and run in SDS running buffer for 1.5 h at 125 V. The separated proteins were transferred onto a nitrocellulose membrane (Amersham Protran, GE Healthcare) by wet-blot electrophoresis for 2 h at 300 mA. Antibodies are listed in Supplementary Table 3.

### Flow cytometry

Cells were stained with fluorochrome-labeled antibodies according to manufacturer information (listed in Supplementary Table 3). To block free Fc receptors murine CD16/32 or human Fc Receptor Binding Inhibitor Polyclonal Antibody (eBioscience) were applied. Dead cells were excluded by DAPI (1 µg/ml) (Sigma Aldrich) staining. General gating strategies for surface marker expression and viability assessment are shown in Supplementary Fig. 5. Flow Cytometry was performed using a FACS Canto II cytometer (BD Bioscience). Data were analyzed with the FlowJo^TM^ software version 10.7.1 (BD Bioscience).

### Intracellular staining

To evaluate AKT phosphorylation, anti-phosho AKT (Ser473 (D9E) Rabbit mAb (PE conjugate, #5315 CST)) or matched isotype control were used. Upon treatment for 3 min with 3AC or control, MEC-1 cells were washed in PBS, fixed with 4% formaldehyde, and permeabilized with methanol (90% final concentration) according to manufacturer’s instructions (CST).

### SHIP1 knockdown and SHIP inhibitor treatment in vivo

To inhibit SHIP1 in vivo mice were treated with 20 mg/kg 3AC (MoBiTec Molecular Biotechnology) or the vehicle, Hydroxypropylcellulose (Klucel, Sigma Aldrich) via intraperitoneal injections. NSG mice were xenotransplanted with 2 × 10e6 MEC-1 or MEC-1 Luc CLL cells via intravenous injection into the tail vein and treatment started 2 days after transplantation. Engraftment and progression of MEC-1 Luc cells were followed by IVIS bioimaging. Mice were sacrificed upon clear signs of disease, comprising neurological symptoms and weight loss. Patient-derived xenotransplanted cells (4 × 10e7 i.v., 4 × 10e7 i.p.)^75^ were treated with 3AC starting at day 1 after transplantation for 4–8 daily doses. Peripheral blood (PB) analysis determined the endpoint and organs were harvested when <10% human CLL cells were detectable in the PB. In the murine TCL1tg model, 3AC treatment was initiated when PB of transplanted mice confirmed a CLL positive population <5% or when CLL content was on average 50% in the peripheral blood. Mice were treated daily, in different treatment cycles as described in Supplementary Fig. 3. Organs were harvested at experimental termination and analyzed for CLL content by flow cytometry. Samples for histology were fixed in 4% PFA.

### Histology

Murine spleens were fixed in 10% neutral buffered formalin (for 48 h) and then dehydrated and embedded in Paraffine (Leica ASP 300S) according to routine methods. To detect and analyze infiltration with neoplastic cells, blocks were cut (2 μm thickness) and stained with Hematoxylin-Eosin or anti-human CD20cy antibody (Agilent, clone L26, 1:2000). CD20 IHC was performed on a Leica BondRxm using a Polymer Refine detection kit. All slides were scanned with a Leica AT2 scanning system with ×40 magnification. HE stainings and IHCs were evaluated by a board-certified veterinary pathologist and the amount of CD20 positive neoplastic cells in the spleen was analyzed by using the Aperio PositivePixelCount v9. The number of positive pixels was calculated per mm^2^ and the results between the groups (3AC-treated vs. control) were visualized and statistics (Mann–Whitney *U* test) were calculated with IBM SPSS statistics 25.

### Bioimaging

MEC-1 Eco cells were transduced with firefly luciferase retrovirus and selected with blasticidin for bioluminescence imaging^14^. 1 h after transplantation of MEC-1 Luc cells into NSG mice, 2 mg Luciferin were i.p. administered per mouse. After a 10 min integration time, luminescence in mice was captured using the IVIS Lumina imaging system (Perkin Elmer) and exposed for 10 s, 1 min, and 5 min. The Living Image Software (Perkin Elmer) was used for analysis.

### SHIP1 activity assay

Whole-cell lysates of 1 × 10^8^ primary cells of CLL patients (*n* = 4) and isolated B cells of healthy donor-derived samples (*n* = 5) were prepared using NP40 buffer for the SHIP1 inositol phosphatase activity assay^29^. H1299 cells that lack endogenous SHIP1 expression were used to lentivirally overexpress *wt* SHIP1 or the AML-derived R673Q variant of SHIP1 and served as a positive and negative control, respectively. 1 mg of each lysate was used for immunoprecipitation of SHIP1 using a mouse monoclonal antibody (SHIP1 P1C1, Santa Cruz Biotechnology), which had been coupled to G sepharose beads. For the assay, the buffer was changed from NP40 buffer to phosphatase assay buffer and the beads containing immunoprecipitated SHIP1 were separated in four parts per sample. Three parts were used for the three assay replicates and the remaining part for western blot analysis to evaluate the amount of immunoprecipitated SHIP1. For the assay, a commercially available phosphate assay kit (Bioassay systems) was used with 20 μM f.c. ci8-Phosphatidylinositol-3,4,5-trisphosphate as substrate in a final volume of 400 μl. The sample was preincubated for 5 min at 37 °C after which the substrate was added. 90 μl aliquots were taken after 5, 20, 60, and 120 s, and the reaction was stopped by the addition of 31.5 μl of 0.1 M EDTA. The amount of released phosphate was evaluated photometrically as instructed in the assay kit. To determine the enzyme activity, the initial change in nmol phosphate (5–20 s)/min was calculated.

### Cell stimulation and inhibitor treatment

Murine TCL1tg splenocytes (>70% CLL), primary patient-derived peripheral blood CLL cells (>70% CLL) and healthy peripheral blood MACS-isolated B cells were seeded at 200,000 cells/well in 96-well u-bottom plates or 500,000 cells in 24-well plates and treated with 3AC (Echelon Bioscience) (or K118 (Echelon Biosciences) when indicated) up to 48 h. Viability was analyzed by flow cytometry. In combinatorial treatment experiments with the inhibitors GSK-843 (Selleckchem; 1 μM), Z-IETD (BD Bioscience; 10 μM), Emricasan (Selleckchem; 5 μM), AZD-5363 (Toric Bioscience; 5 μM), NEC1s (BioVision; 30 μM), NSA (Cayman Chemical; 2μM) cells were pre-treated for 1 h, followed by addition of 5 μM 3AC for 48 h. Cell lines (MEC-1, EHEB, Bal17, SUDHL6, BAJB) were synchronized by cell count and resuspended at 1 × 10^6^ cells/ml in a fresh culture medium 1 day prior to treatment. Cells were then treated with 3AC at 100,000 cells/well in 96-well u-bottom plates or 500,000 cells/well in 24-well plates.

### ATP measurement

To measure the release of ATP, patient-derived CLL cells were treated for 4 h with 3AC as described and supernatants were analyzed with the ATP Enliten Kit (Promega), according to manufacturer’s instructions. Similarly, supernatants of puromycin-selected (shRNA expressing, d6) or GFP-sorted (EV, myrAKT expressing, d5) MEC-1 cells were collected and analyzed with the Cell Titer Glo Kit (Promega) according to the manufacturer’s instructions. For detection of luminescence the GloMax® Discover platereader was used (Promega).

### Specific cell death

DAPI negative cells were defined as viable cells via flow cytometry. Percentage of specific cell death was calculated by this formula: specific cell death (%) = 100 × (% dead cells − % baseline dead cells)/(100 − % baseline dead cells).

### Genetic knockdown

shRNA SHIP1 knockdown1 (KD1) and shRNA SHIP1 knockdown2 (KD2) as well as scramble (scr) were purchased from Sigma Aldrich and subcloned into pLKO.1. For the generation of SHIP1 KD MEC-1 cell lines, MEC-1 Eco and MEC-1 Eco/Luc cells were transduced with scramble or 2 constructs of SHIP1-targeting shRNA (KD1, KD2) concentrated lentivirus (pCMV delta R8.2: Addgene #12263, RRID: Addgene_12263), Phit123: kindly provided by Markus Müschen). 5 days after spin infection, Puromycin selection was initiated with 1 μg/ml for 3 days. Cell viability before and after Puromycin selection was assessed by flow cytometric DAPI exclusion and by total cell count. SHIP1 knockdown was confirmed by western blotting. 1 × 10^6^ shSHIP1 KD1, KD2 MEC-1 cells, and shScramble control-containing MEC-1 cells were transplanted into NSG mice and CLL engraftment and progression was followed by bioimaging as described above. Bone marrow and spinal cord cells were isolated from sacrificed mice and MEC-1 (GFP^+^, CD19^+^) cells were FACS sorted and SHIP1 expression levels were analyzed by qPCR.

### Genetic knockout

To generate SHIP1 knockout (KO) CLL cells, MEC-1 Eco cells were transduced with *INPP5D*-targeting guide RNAs (5 different sgRNAs, listed in Supplementary Table 2) and pMIG-Cas9 containing retrovirus^14^. After 48 h from transduction cells were selected with 1 μg/ml of puromycin for 3 days. The selected cells were seeded as single colonies in 96-well plates by FACS sorting. After 3–4 weeks of culture, cells derived from each colony were used to assess SHIP1 knockout by western blotting and genomic sequencing of the sgRNA target region (Amplification and sequencing primer are listed in Supplementary Table 2). 2 × 10^6^ SHIP1 KO (3 independent clones) and SHIP1 wild-type (2 independent clones) MEC-1 cells were transplanted into NSG mice. The mice were sacrificed upon clear signs of disease, comprising neurological symptoms and weight loss. Prior to transplantation, all cells were kept overnight at 1 × 10^6^/ml in fresh media, and equal viability was confirmed prior to injection.

### Competition assay

The growth behavior of MEC-1 SHIP1 KO cells was analyzed by competition assays. 500,000 GFP-positive knockout cells were mixed with equal amounts of MEC-1 wild-type, GFP-negative cells. The percentage of GFP-expressing cells was followed by flow cytometric analysis over time.

### RNA preparations/RNASeq

For bulk 3′-sequencing of poly(A)-RNA (RNASeq), viable GFP^+^ MEC-1 cells on day 7 post-transduction with pMIG-empty (EV) or pMIG-myrAKT1 were sorted and RNA was extracted from whole-cell lysates via RNeasy Mini Kit (Qiagen, Hilden, Germany). Barcoded cDNA of each sample was generated with a Maxima RT polymerase (ThermoFisher) using oligo-dT primer containing barcodes, unique molecular identifiers (UMIs), and an adapter. 5′ ends of the cDNAs were extended by a template switch oligo (TSO) and after pooling of all samples, full-length cDNA was amplified with primers binding to the TSO-site and the adapter^76^. cDNA was fragmented and TruSeq-Adapters ligated with the NEBNext® Ultra™ II FS DNA Library Prep Kit for Illumina® (NEB) and 3′-end-fragments were finally amplified using primers with Illumina P5 and P7 overhangs. In comparison to Parekh et al., the P5 and P7 sites were exchanged to allow sequencing of the cDNA in read1 and barcodes and UMIs in read2 to achieve a better cluster recognition. The library was sequenced on a NextSeq 500 (Illumina) with 75 cycles for the cDNA in read1 and 16 cycles for the barcodes and UMIs in read2.

For analysis, gencode gene annotations v28 and the human reference genome GRCh38 were derived from the Gencode homepage (EMBL-EBI). Drop-Seq tools v1.12^77^ were used for mapping raw sequencing data to the reference genome. Shown experiments (myrAKT1 overexpression and Ship1 knockdown with the respective controls) were separately processed. The resulting UMI filtered count matrices were imported into R v3.4.4. CPM (counts per million) values were calculated for the rawdata and genes having a mean CPM value less than 1 were removed from the dataset. Prior differential expression analysis with DESeq2 v1.18.1^78^, dispersion of the data was estimated with a parametric fit using an univariate model where treatment was specified as an independent variable. The Wald test was used for determining differentially regulated genes between treaetments within each individual patient and shrunken log2-fold changes were calculated afterwards. A gene was determined as differentially regulated if the absolute apeglm shrunken log2-fold change was at least 1 and the adjusted *p*-value was below 0.01.

GSEA v4.0.3^49^ was performed in the weighted Preranked mode, where either the Wald test-statistic or the apeglm shrunken fold change was used as ranking metric. All genes tested for differential expression were used for GSEA analysis, with genesets from MsigDB v7.1^79^ where used for testing. A pathway was considered to be significantly associated with treatment if the FDR value was below 0.05. Rlog transformation of the data was performed for visualization and further downstream analysis. Genes of Oxphos pathway contributing most to the NES score of the GSEA are displayed as Heatmap. Heatmap shows *z*-transformed expression data. Raw sequencing data are available from the European Nucleotide Archive under the accession number PRJEB38070.

### Oxidative stress

Cellular and mitochondrial ROS levels were determined by flow cytometry using CellROX^®^ Orange (ThermoFisher Scientific). To analyze the impact of ROS in therapy-induced cell death, CLL cells were co-treated with 3AC in presence of ROS scavengers MitoTEMPO (10 μM) (Merck) or NAC (2 mM) (*N*-acetyl-cysteine) (Sigma Aldrich) for 24–48 h.

### Extracellular flux analysis

Oxygen consumption rate (OCR) and extracellular acidification rate (ECAR) were measured using a Seahorse XFe96 Flux Analyzer with the XF Cell Mito Stress Test Kit and XF Glycolysis Stress Test Kit (Agilent) according to the manufacturer’s instructions. All compounds and materials were obtained from Agilent.

### Statistical analysis

Statistical significance was analyzed with paired or unpaired two-tailed Student’s *t*-test, ordinary one-way ANOVA or log-rank (Mantel–Cox), using Prism Version 7.0, Graphpad Software Inc., as indicated.

### Reporting summary

Further information on experimental design is available in the Nature Research Reporting Summary linked to this paper.

## Supplementary information

Supplementary Information

Description of Additional Supplementary Files

Supplementary Data 1

Reporting Summary

## Data Availability

The RNA sequencing data referenced during the study are available in a public repository from the European Nucleotide Archive (ENA) under the accession code PRJEB38070. INPP5D mutations in CLL can be found on cBioPortal, freely available. All the other data supporting the findings of this study are available within the article and its Supplementary Information files and from the corresponding author upon reasonable request. Source data are provided with this paper.

## Source data

Source Data

## Code availability

All codes used for RNAseq analysis are available upon reasonable request. Source data are provided with this paper.
