# Supplementary material for: Targeted PI3K/AKT-hyperactivation induces cell death in chronic lymphocytic leukemia
Source: Nat Commun. 2021 Jun 10;12:3526. doi: 10.1038/s41467-021-23752-2 (PMC8192787; doi:10.1038/s41467-021-23752-2)
Supplement: Supplementary file 1 — Supplementary Information [file 41467_2021_23752_MOESM1_ESM.pdf]

Supplementary Figure 1

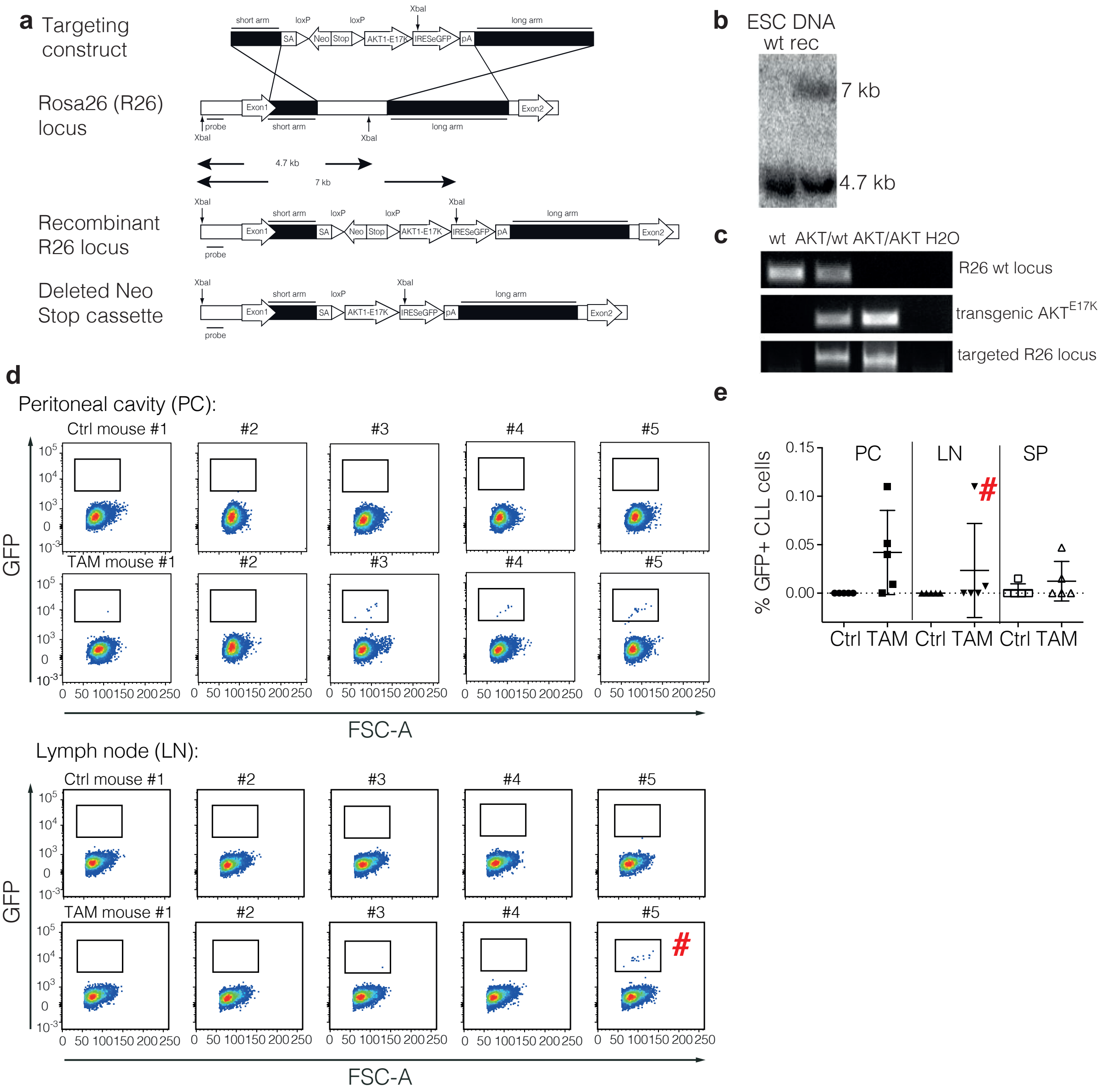

Supplementary Figure 1: a) The targeting construct consists of a short and a long arm homologous to the murine Rosa26 locus, a splice acceptor site (SA), two loxP sites flanking the Neomycin (Neo) Stop cassette, the human AKT1^{E17K} cDNA sequence (codon-optimized for expression in mouse), and the IRES-eGFP sequence followed by a polyadenylation signal (pA). Homologous recombination takes place between Exon 1 and 2 of the Rosa26 locus. The XbaI restriction sites, the resulting fragment sizes for the wildtype Rosa26 (4.7 kb) and the recombinant locus (7 kb), and the binding site for the Southern probe are indicated. Cre expression mediates deletion of the Neomycin-Stop cassette and subsequent expression of AKT1^{E17K} and eGFP. b) Murine embryonic stem cells were electroporated with the linearized targeting construct, plated on feeder cells and selected with Neomycin. Genomic DNA of colonies was prepared and analyzed by Southern Blot after digestion with XbaI. The band at 4.7 kb represents the wildtype Rosa26 locus. The successfully targeted clone shows an additional band at 7 kb. Representatively, one wildtype and recombinant clone are depicted. c) PCRs, specific for the Rosa26 wildtype locus, the transgenic (not endogenous) AKT1^{E17K} and the targeted Rosa26 locus, performed on genomic tail DNA were used to identify germline transmission in the offspring of chimeric mice. d) Single cell suspensions isolated from the respective organs of wt mice transplanted with CLL containing the AKT1^{E17K}

Mb1^{CreERT2} transgenes, 8 weeks post TAM application were pregated for CLL cells via CD19+CD5+ expression and analyzed for the percentage of GFP+ cells. The LN of one mouse revealed a GFP+ population (indicated by the red #), comprising less than 0.2% of CLL cells 8 weeks after TAM application. e) Summary of percent GFP^{+} cells in the indicated organs 8 weeks after TAM application (referring to experimental data shown in Figure 1h-m). Data are presented as individual values and mean values +/- standard deviation (SD).

Supplementary Figure 2

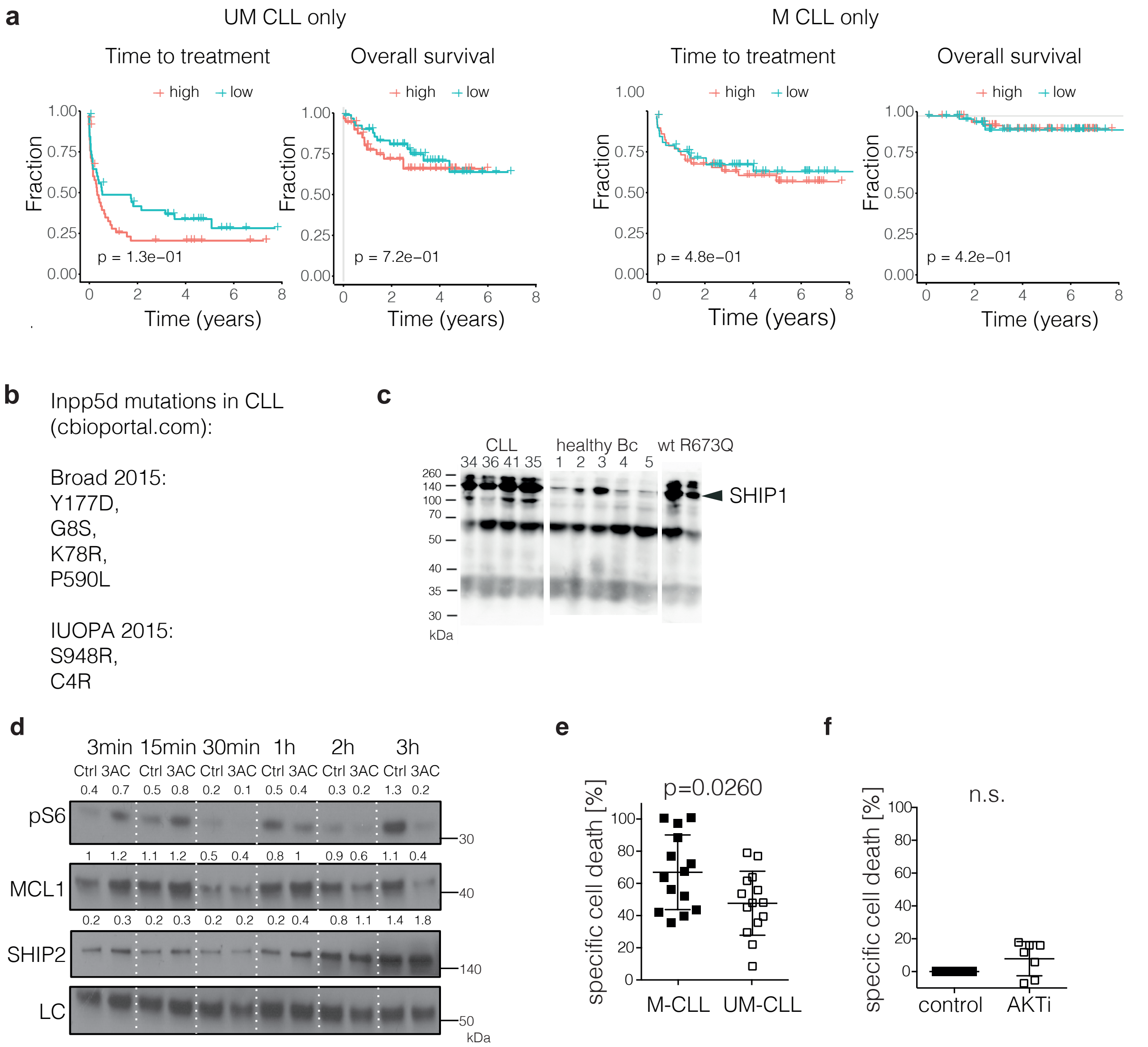

Supplementary Figure 2: a) Kaplan Meier analysis of UM-CLL (n=95, left panel) and M-CLL (n=113, right panel) patients in respect to their INPP5D mRNA expression levels using the median expression value as cut-of. p values were calculated using cox model with INPP5D expression as continuous variable. b) List of identified gene alterations by cbioportal.org in the INPP5D locus of CLL patients. c) Immunoblot analysis of SHIP1 expression levels of the samples subjected to the SHIP1 phosphatase assay shown in Figure 2c. Numbers indicated reflect the sample IDs; clinical data of the CLL samples is listed in Suppl. Table 1. H1299 cells lentivirally transduced with either SHIP1 WT or R673Q served as internal positive and negative controls for the assay. The arrow indicated the band that corresponds to the 145kDa SHIP1 size. d) Specific cell death upon 3AC treatment of primary

CLL samples is shown for the mutated (M-CLL) and unmutated (UM-CLL) subsets. Data are presented as individual values and mean values +/- SD. Statistical significance was assessed by a two-tailed unpaired Student's t test. e) Time course analysis of S6 phorphorlyation, MCL1, and SHIP2 expression upon 3AC treatment in MEC-1 cells. Numbers indicate quantification relative to the loading control Tubulin A (TubA). Representative analysis for 2 independent experiments. f) Primary CLL cells were incubated with the AKT inhibitor AZD-5363 (5 μM) for 24h and the specific cell death was calculated as determined in Figure 2e. Data are presented as individual values and statistical significance was assessed by a two-tailed paired Student's t test. Significance values are depicted in the graph; (n.s.) not significant.

Supplementary Figure 3

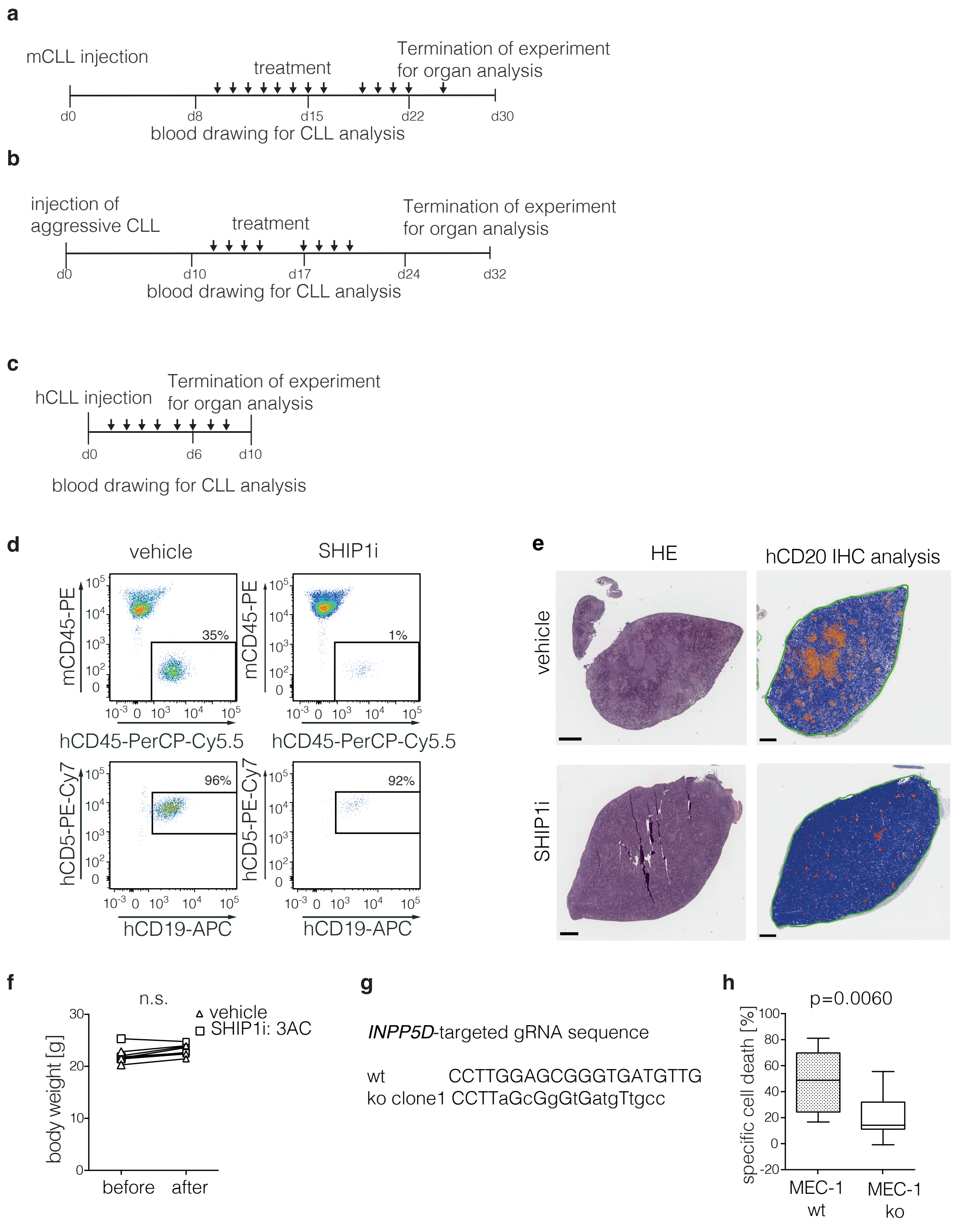

Supplementary Figure 3: a) Treatment schedule of indolent murine CLL *in vivo.* b) Treatment schedule of aggressive murine CLL *in vivo*. c) Treatment schedule of patient-derived xenograft experiment with the SHIP1 specific inhibitor 3AC. d) Gating strategy for the analysis of primary CLL cell content in the spleen of NSG mice upon SHIP inhibitor treatment. e) left: HE staining of spleen histology samples; right: human CD20 IHC analysis

markup for quantification is shown; scale bars represent 500 µm. f) Weight of animals prior and after SHIP1 inhibitor 3AC or vehicle (Klucel/H_2O) treatment *in vivo*. Data are presented as individual values and statistical significance was assessed by a two-tailed paired Student's t test. g) Representative sequencing result of a confirmed SHIP1 knockout MEC-1 clone. Mismatches compared to wt SHIP1 are depicted in lower case. h) MEC-1 control (MEC-1 wt; n=4) and SHIP1 knockout clones (MEC-1 ko; n=7) were incubated with 10 µM 3AC for 24h and analyzed for viability by flow cytometry (DAPI exclusion), summary of 2 independent experiments. Data are presented as box plots indicating median (middle line), 25th, 75th percentile (box) and minimum and maximum (whiskers). Statistical significance was assessed by a two-tailed unpaired Student's t test.

Supplementary Figure 4

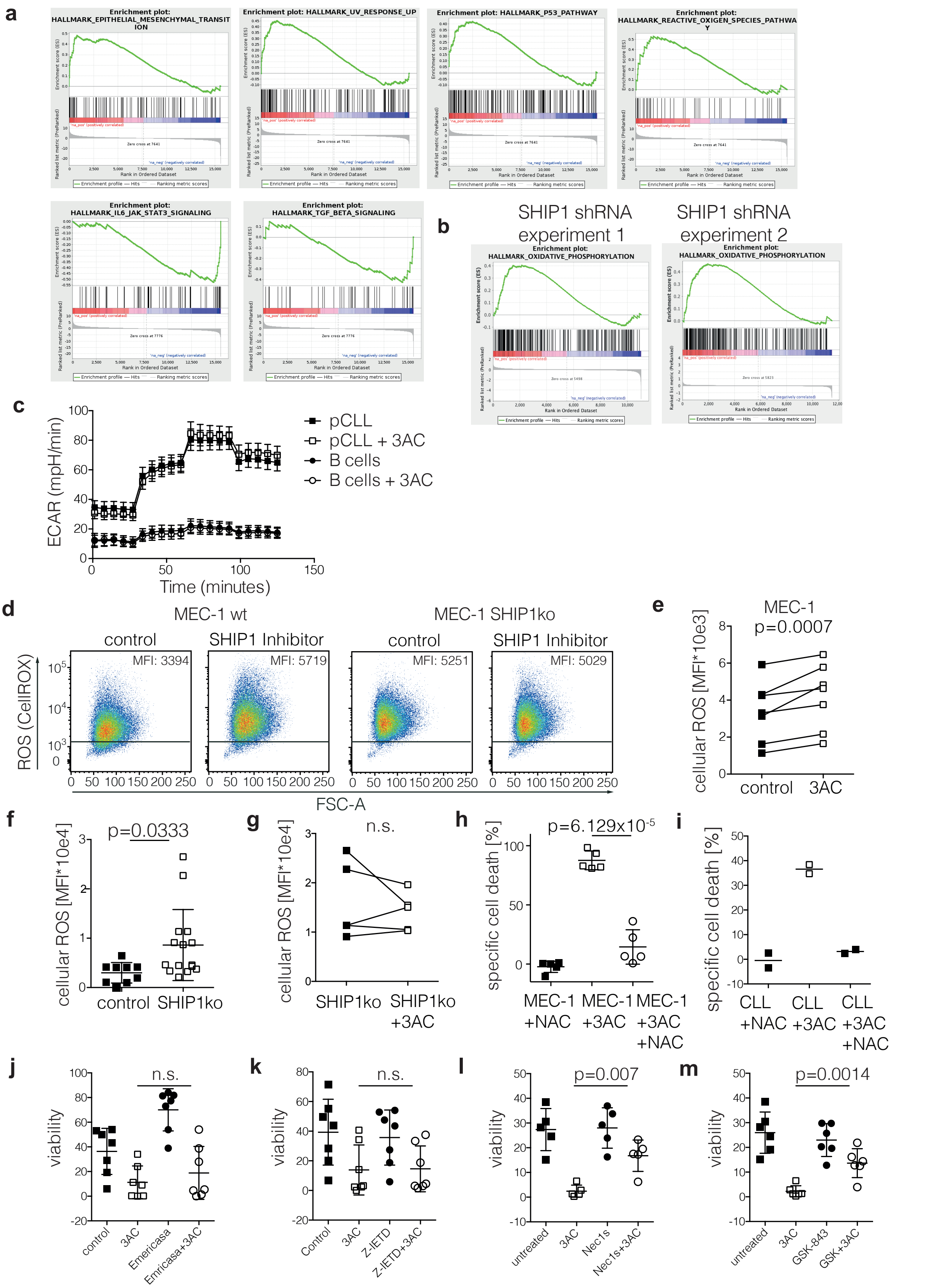

Supplementary Figure 4: a) Gene set enrichment analysis (GSEA) results of altered signatures upon myrAKT1 expression of two independent experiments are depicted. b) GSEA results for the association with 'oxidative phosphorylation' is shown upon SHIP1 downregulation by shRNA for 2 independent experiments, Enrichment Score (ES) 0.4049174 (left) and 0.4637735 (right); Normalized Enrichment Score (NES) 1.4929111 (left) and

1.5490396 (right); Nominal p-value 0.0041841003 (left) and 0.001197 (right); FDR q-value 0.02699195 (left) and 0.10192727 (right); FWER p-Value 0.193 (left) and 0.0.413 (right); c) Extracellular acidification rate (ECAR) was measured in primary CLL samples and B-cells derived from healthy donors (n=6, respectively) treated for 1h with 5 μM 3AC or control using a Seahorse XFe96 Flux Analyzer with the XF Glycolysis Stress Test Kit (Agilent) according to the manufacturer's instructions. d) Representative FACS analysis ROS levels were measured by CellROX staining and flow cytometric analysis in MEC-1 wt or knockout clones; e) MFI summary of ROS levels measured by CellROX staining and flow cytometric analysis in MEC-1 wt after 4h treatment with 5 μM 3AC, n=7; pooled analysis from 3 independent experiments. Data are presented as individual values and statistical significance was assessed by a two-tailed paired Student's t test. f) Summary of MFI after CellROX staining and flow cytometric analysis in MEC-1 wt (n=9) and SHIP1 knockout clones (n=15) indicate higher levels of ROS in SHIP1 knockout cells; pooled analysis from 3 independent experiments. Data are presented as individual values and mean values +/- SD. Statistical significance was assessed by a two-tailed unpaired Student's t test. g) Treatment of SHIP1 knockout clones for 4h with 5 μM 3AC did not further increase ROS levels as shown by matched pair analysis of MFI, after CellROX staining and flow cytometric analysis; n=5; pooled analysis from 3 independent experiments. Data are presented as individual values and statistical significance was assessed by a two-tailed paired Student's t test. h) Specific cell death for MEC-1 cells treated with 3AC in presence and absence of ROS scavenger N-acetyl-cysteine (NAC, 2 mM) is shown, summary of 5 independent experiments. Data are presented as individual values and statistical significance was assessed by a two-tailed paired Student's t test. i) Specific cell death for 5 primary CLL samples treated with 3AC in presence and absence of ROS scavenger N-acetyl-cysteine (NAC, 2 mM) is shown. Data are presented as individual values measured in 2 independent experiments. k-m) Viability of primary CLL samples treated with the SHIP1 inhibitor (SHIP1i) 3AC or the combination of 3AC with the Caspase inhibitor (PanCaspi) Emricasan (**j**, n=7), the Caspase 8 inhibitor (Casp8i) Z-IETD (**k**, n=8), the RIP1 inhibitor (RIP1i) NEC1s (**l**, n=5) or the RIP3 inhibitor (RIP3i) GSK-843 (**m**, n=7)**.** Data refers to Fig. 6 a-d. Data are presented as individual values and mean +/-SD; statistical significance was assessed by a two-tailed paired Student's t test.

Supplementary Figure 5

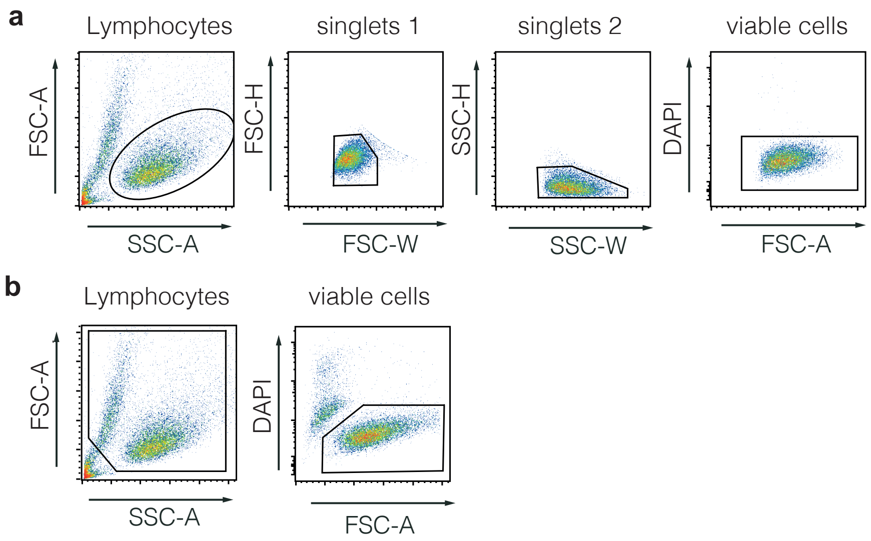

Supplementary Figure 5: a) General gating strategy for surface and intracellular marker expression is shown (in Fig. 1a, b, d, e, g, h, l, m; Fig2d; Fig. 3a-f; Fig4h; Fig. 5f; Fig. 6e; Suppl. Fig. S1d, e, Suppl. Fig. S3d, Suppl. Fig. S4d-g; b) Gating strategy to determine cell viability by flow cytometry is depicted (used in Fig. 1f; Fig.2 e-h; Fig. 4b, h; Fig. 5g, h; Fig 6a-d; Suppl. Fig. S2e, f; Suppl. Fig. S3h, S4h-m).

Supplementary Table 1: CLL patient's characteristics

Sample ID	Mutation or ZAP70 status	% lymphocytes	WBC	Del13q14	Del17p13	TP53 mutation
CLL1	M[1)]	46	185.9	yes	yes	no
CLL2	M	93	147.4	yes, biallelic	no	no
CLL3	UM[2)]	96	139.9	yes	no	no
CLL4	M	56	9.84	yes, biallelic	no	no
CLL5	UM	88	46.3	no	yes	yes
CLL6	M	86	50.7	yes	no	no
CLL7	M	97	88.7	no	no	yes
CLL8	M	99	83.9	yes	no	no
CLL9	M	98	93.9	yes	no	no
CLL10	M	76	31.8	yes	no	no
CLL11	UM	98	76.4	yes	no	no
CLL12	M	33	6.8	yes, biallelic	no	no
CLL13	UM	93	107.5	yes	no	no
CLL14	UM	96	85.4	yes	no	no
CLL15	UM	97	205.9	no	no	no
CLL16	M	97	191.9	no	no	yes
CLL17	UM	93	130.9	yes	yes	n.a.[3)]
CLL18	M	86	59.6	no	no	no
CLL19	UM	85	65.4	no	no	no
CLL20	UM	90	117.3	no	no	no
CLL21	M	96	n.a.	yes	no	no
CLL22	M	91	n.a.	yes	no	no
CLL23	M	93	n.a.	yes	no	no
CLL24	M	81	n.a.	yes	no	yes
CLL25	M	n.a.	n.a.	n.a.	n.a.	no
CLL26	UM	85	n.a.	no	no	no
CLL27	UM	73	n.a.	no	no	no
CLL28	UM	94	n.a.	no	no	no
CLL29	UM	92	n.a.	no	no	no
CLL30	UM	97	n.a.	no	no	no
CLL31	UM	97	360	yes	no	no
CLL32	M	51	14.7	no	no	no
CLL33	M	77	28.2	no	no	no
CLL34	UM	91	73.4	no	yes	no
CLL35	M	93	92.8	yes	no	no
CLL36	UM	n.a.	n.a.	no	no	yes
CLL37	ZAP70 neg[4)]	93	43.4	yes	yes	no
CLL38	ZAP70 neg	58	13.5	n.a.	n.a.	n.a.
CLL39	n.a.	65	24.4	n.a.	n.a.	n.a.
CLL40	ZAP70 pos[5)]	n.a.	27.7	n.a.	n.a.	n.a.
CLL41	ZAP70 neg	99	112.7	n.a.	n.a.	n.a.
CLL42	n.d.	97	312.4	yes	yes	n.a.
CLL43	n.d.	97	320.9	yes	yes	n.a.
CLL44	ZAP70 pos	33	12.8	n.a.	n.a.	n.a.

[1)]M = mutated IgV_H, [2)]UM = unmutated IgV_H, [3)]n.a. = not available, [4)]ZAP70 pos = positive, [5)]ZAP70 neg = negative

Supplementary Table 2: Sequences of primers and guide RNAs

**Genotyping Primers:**	
TCL1 FW	AGTGGTAAATATAGGGTTGTCTACACG
TCL1 RV	CCCGTAACTGTAACCTATCCTTTA
Mb1CreERT2 FW	ACAAAGGGGAAAGGGAAGAA
Mb1CreERT2 RV	CATGTTTAGCTGGCCCAAAT
AKT E17K FW	TACAAGGACGACGACGACAAG
AKT E17K RV	CTGCCGCTTCTGAAGTCCA

**qPCR Primers:**	
SHIP1 FW	AGTCAGCGGGATGTTTCTTG
SHIP1 RV	GCTGGAGGAAGAGGACACAG
GAPDH FW	GTCGCTGTTGAAGTC
GAPDH RV	GAAACTGTGGCGTGA

Inpp5d gRNAs

human SHIP1: targeted gRNA 5'-3' sequence

hSHIP1 guide 1 FW: caccgCAGGCATTGCAAACACACTG
hSHIP1 guide 1 RV: aaacCAGTGTGTTTGCAATGCCTGc

hSHIP1 guide 2 FW: caccgCTGACACACCACGTGCACCA
hSHIP1 guide 2 RV: aaacTGGTGCACGTGGTGTGTCAGc

hSHIP1 guide 3 FW: caccgGCTCTGGAACATCCGCATCG
hSHIP1 guide 3 RV: aaacCGATGCGGATGTTCCAGAGCc

hSHIP1 guide 4 FW: caccgGTGGCTGTTGACGAACCCTA
hSHIP1 guide 4 RV: aaacTAGGGTTCGTCAACAGCCACc

hSHIP1 guide 5 FW: caccgCAACATCACCCGCTCCAAGG
hSHIP1 guide 5 RV: aaacCCTTGGAGCGGGTGATGTTGc

Amplification Primer for DNA Inpp5d KO confirmation:

hInpp5d amplif Exon 1 FW: ggcctcgagccaccATGGTCCCCTGCTGGAACCATG
hInpp5d amplif Exon 1 RV: GAACAACCCATCTCAAAGCTGGG

hInpp5d amplif Exon 13 FW: GATTACAGACAGGATACCCCATACCC
hInpp5d amplif Exon 13 RV: CTTTCCTGCAAAAGTTGGCCAATGC

hInpp5d amplif Exon 14 FW: TTGCCAGCTCCTCACTCACTG
hInpp5d amplif Exon 14 RV: CAAAGGCAGGAGCTGAGC

hInpp5d amplif Exon 18 FW: GAAGTAGTCAGGAGGATATGTGAGG
hInpp5d amplif Exon 18 RV: GACACATCAACAGACTGAAGCCC

Sequencing Primer for amplified Inpp5d KO DNA segments:

hInpp5d Exon 1 guide RV Seq: GAACAACCCATCTCAAAGCTGGG
hInpp5d Exon 13 guide FW Seq: GATTACAGACAGGATACCCCATACCC
hInpp5d Exon 14 guide FW Seq: TTGCCAGCTCCTCACTCACTG
hInpp5d Exon 18 guide RV Seq: GACACATCAACAGACTGAAGCCC

Supplementary Table 3: Antibodies (further information in Reporting Summary)

Flow cytometry

Anti-human	CD5	PE-Cy7 (L17F12)	BioLegend
Anti-human	CD19	APC (HIB19)	BioLegend
Anti-human	CD45	PerCP-Cy5.5 (2D1)	Invitrogen
Anti-mouse	CD19	APCeFluor780 (1D3)	eBioscience
Anti-mouse	CD19	AmCyan (6D5)	BioLegend
Anti-mouse	CD45	PE (30-F11)	Invitrogen
Anti-mouse	CD5	PE (53-7.3)	eBioscience
Anti-mouse	CD5	APC (53-7.3)	eBioscience
Anti-mouse	CD4	BD Horizon V500 (RM4-5)	BD Bioscience
Anti-mouse	CD8	APC/Cy7 (53-6.7)	BioLegend
Anti-mouse	CD62L	FITC (Mel-14)	BioLegend

Western blot

phospho-Akt Ser473	Cell Signaling Technology	#9271
AKT	Cell Signaling Technology	#9272
phospho-eIF2α	Cell Signaling Technology	#3398S
eIF2α	Cell Signaling Technology	#5324S
phospho-SHIP-1 Tyr1020	Cell Signaling Technology	#3941
SHIP-1	Cell Signaling Technology	#2727
MCL-1	Cell Signaling Technology	#94296
SHIP2	Cell Signaling Technology	#2730
P-S6	Cell Signaling Technology	#14733
Tubulin A	Cell SignalingTechnology	#2125
mouse IgG HRP-linked (horse polyclonal IgG)	Cell Signaling Technology	#7076
rabbit IgG HRP-linked (goat polyclonal IgG	Cell Signaling Technology	#7074
HMGB-1	Abcam	ab18256
DHX9	Proteintech Europe	17721-1-AP
ActB	Proteintech Europe	60008-I-Ig
