# Supplementary material for: Targeted PI3K/AKT-hyperactivation induces cell death in chronic lymphocytic leukemia
Source: Nat Commun. 2021 Jun 10;12:3526. doi: 10.1038/s41467-021-23752-2 (PMC8192787; doi:10.1038/s41467-021-23752-2)
Supplement: Supplementary file 2 — Description of Additional Supplementary Files [file 41467_2021_23752_MOESM2_ESM.pdf]

Description of Additional Supplementary Files

Supplementary Data 1:

The GSEA report of 2 independent experiments of MEC-1 cells transduced with pMIG-myrAKT versus empty vector (pMIG-EV) control is listed, each with up- and downregulated signatures. Raw sequencing data is available from the European Nucleotide Archive under the accession number PRJEB38070.
