# Supplementary material for: Targeted PI3K/AKT-hyperactivation induces cell death in chronic lymphocytic leukemia
Source: Nat Commun. 2021 Jun 10;12:3526. doi: 10.1038/s41467-021-23752-2 (PMC8192787; doi:10.1038/s41467-021-23752-2)
Supplement: Supplementary file 4 — Reporting Summary [file 41467_2021_23752_MOESM4_ESM.pdf]

nature research

Corresponding author(s): Maike Buchner

Last updated by author(s): Mar 18, 2021

# Reporting Summary

Nature Research wishes to improve the reproducibility of the work that we publish. This form provides structure for consistency and transparency in reporting. For further information on Nature Research policies, see our Editorial Policies and the Editorial Policy Checklist.

## Statistics

For all statistical analyses, confirm that the following items are present in the figure legend, table legend, main text, or Methods section.

| n/a | Confirmed | |
|---|---|---|
| ☐ | ☒ | The exact sample size (*n*) for each experimental group/condition, given as a discrete number and unit of measurement |
| ☐ | ☒ | A statement on whether measurements were taken from distinct samples or whether the same sample was measured repeatedly |
| ☐ | ☒ | The statistical test(s) used AND whether they are one- or two-sided<br>*Only common tests should be described solely by name; describe more complex techniques in the Methods section.* |
| ☐ | ☒ | A description of all covariates tested |
| ☐ | ☒ | A description of any assumptions or corrections, such as tests of normality and adjustment for multiple comparisons |
| ☐ | ☒ | A full description of the statistical parameters including central tendency (e.g. means) or other basic estimates (e.g. regression coefficient) AND variation (e.g. standard deviation) or associated estimates of uncertainty (e.g. confidence intervals) |
| ☐ | ☒ | For null hypothesis testing, the test statistic (e.g. *F*, *t*, *r*) with confidence intervals, effect sizes, degrees of freedom and *P* value noted<br>*Give P values as exact values whenever suitable.* |
| ☒ | ☐ | For Bayesian analysis, information on the choice of priors and Markov chain Monte Carlo settings |
| ☒ | ☐ | For hierarchical and complex designs, identification of the appropriate level for tests and full reporting of outcomes |
| ☒ | ☐ | Estimates of effect sizes (e.g. Cohen's *d*, Pearson's *r*), indicating how they were calculated |

*Our web collection on statistics for biologists contains articles on many of the points above.*

## Software and code

Policy information about availability of computer code

| | |
|---|---|
| Data collection | Flow Cytometry: FACSDiva Version 8.0.1 (BD Biosciences) for FACS CantoII und FACS Aria III; RNAseq: NextSeq Control Software Version 2.2.0.4 (Illumina); Bioimaging: Living Image Software Version 4.4 (Perkin Elmer); Metabolism: Seahorse Wave Software Version 2.6.1 (Aglient), ImageJ Version 1.50i |
| Data analysis | Flow Cytometry: FlowJoTM software version 10.6.2 (BD Bioscience); Data visualization and statistical analysis: Prism Version 7.0 (Graphpad Software Inc.); RNAseq: Dropseq Tools Version 1.12; R Version 3.4.4; DESeq2 Version 1.18.1; GSEA Version 4.0.3; MsigDB Version 7.1 |

For manuscripts utilizing custom algorithms or software that are central to the research but not yet described in published literature, software must be made available to editors and reviewers. We strongly encourage code deposition in a community repository (e.g. GitHub). See the Nature Research guidelines for submitting code & software for further information.

## Data

Policy information about availability of data

All manuscripts must include a data availability statement. This statement should provide the following information, where applicable:

- Accession codes, unique identifiers, or web links for publicly available datasets
- A list of figures that have associated raw data
- A description of any restrictions on data availability

The RNA sequencing data that support the findings of this study have been deposited in the European Nucleotide Archive (ENA) under the accession code PRJEB38070. Source data underlying Figs. 1–6 and Supplementary Figs. 1-5 will be provided as a Source Data file with the paper. All data are available from the authors upon reasonable requests.

# Field-specific reporting

Please select the one below that is the best fit for your research. If you are not sure, read the appropriate sections before making your selection.

- [x] Life sciences
- [ ] Behavioural & social sciences
- [ ] Ecological, evolutionary & environmental sciences

For a reference copy of the document with all sections, see nature.com/documents/nr-reporting-summary-flat.pdf

# Life sciences study design

All studies must disclose on these points even when the disclosure is negative.

| | |
|---|---|
| Sample size | For in vivo experiments, a sample size of n = 5-7 mice was used per experimental group. Sample size was determined based on our previous experience and published data sets (Nat Commun. 2015;6:7951), which is sufficient to generate statistically significant results. No statistical method was used to predetermine sample size. For in vitro experiments, at least three biological replicates (cells from at least 3 CLL donors) were chosen for most of the experiments, except for experiments using the MEC-1 cell line, where the results of at least 3 independent experiments is shown. |
| Data exclusions | No data were excluded from the analyses. |
| Replication | All experimental findings were reliably reproduced as indicated in the figure legends. |
| Randomization | Blood donors for the CLL and heatlhy donor B cells were selected randomly. For in vivo models, TCL1-derived CLL cells and primary CLL donors were randomly selected. Upon detection of CLL in the peripheral blood, mice were assigned to experimental and control groups so that the CLL burden was consistent across the experimental groups. |
| Blinding | Blinding was not performed because the reported outcomes are based on non-subjective measurements. Experiments were designed so that multiple samples were prepared and measured side-by-side in a highly uniform manner. Appropriate controls were always included and treated similar. |

# Reporting for specific materials, systems and methods

We require information from authors about some types of materials, experimental systems and methods used in many studies. Here, indicate whether each material, system or method listed is relevant to your study. If you are not sure if a list item applies to your research, read the appropriate section before selecting a response.

## Materials & experimental systems

| n/a | Involved in the study | |
|---|---|---|
| [ ] | [x] | Antibodies |
| [ ] | [x] | Eukaryotic cell lines |
| [x] | [ ] | Palaeontology and archaeology |
| [ ] | [x] | Animals and other organisms |
| [ ] | [x] | Human research participants |
| [x] | [ ] | Clinical data |
| [x] | [ ] | Dual use research of concern |

## Methods

| n/a | Involved in the study | |
|---|---|---|
| [x] | [ ] | ChIP-seq |
| [ ] | [x] | Flow cytometry |
| [x] | [ ] | MRI-based neuroimaging |

## Antibodies

| | |
|---|---|
| Antibodies used | Anti-human CD5 PE-Cy7 (L17F12) BioLegend Cat. No. 364013; Anti-human CD19 APC (HIB19) BioLegend Cat. No. 302212; Anti-human CD45 PerCP-Cy5.5 (2D1) Invitrogen Cat. No. 45-9459-42; Anti-mouse CD19 APCeFluor780 (1D3) eBioscience Cat. No. 47-0193-82; Anti-mouse CD19 AmCyan (6D5) BioLegend Cat. No. ; Anti-mouse CD45 PE (30-F11) Invitrogen Cat. No. 12-0451-82; Anti-mouse CD5 PE (53-7.3) eBioscience Cat. No. 12-0051-82; Anti-mouse CD5 APC (53-7.3) eBioscience Cat. No. 17-0051-82;<br>phospho-Akt Ser473 Cell Signaling Technology (CST) #9271; AKT CST #9272; phospho-eIF2α CST #3398S, eIF2α CST #5324S, phospho-SHIP-1 Tyr1020 CST #3941, SHIP-1 CST #2727, MCL-1 CST #94296, SHIP2 CST #2730, phospho-S6 CST #14733, Tubulin A CST #2125, mouse IgG HRP-linked (horse polyclonal IgG) CST #7076, rabbit IgG HRP-linked (goat polyclonal IgG) CST #7074, HMGB-1 Abcam ab18256, DHX9 Proteintech Europe 17721-1-AP, ActB Proteintech Europe 60008-I-Ig. Anti-human Fc/anti-mouse Fc; anti-CD20 (histo) |
| Validation | All antibodies used in this study were commercially available and validated by the respective manufacturer for their use in flow cytometry, Western Blot or histology. More detailed information about their specificity and validation are described in the following and can also be found on the manufacturer`s websites.<br><br>Flow cytometry: Human Fc Receptor Binding Inhibitor Polyclonal Antibody (eBioscience, LOT: 2235896, Cat No: 14-9161-73, Specifity: Fc receptor-mediated non-specific binding is blocked by Fc Receptor Binding Inhibitor. THP-1 cells were left untreated or treated with Fc Receptor Binding Inhibitor Antibody. The cells were then stained with different isotype controls (Mouse IgG2a kappa Isotype Control (APC) or Syrian Hamster Isotype Control (APC) or Rat IgG2b kappa Isotype Control (APC)). Histogram analysis of Fc receptor |

treated and isotype treated cells overlayed with untreated autofluorescent cells and did not show a specific signal as compared to Fc receptor untreated, isotype treated cells: https://www.thermofisher.com/antibody/product/Fc-Receptor-Binding-Inhibitor-Antibody-Polyclonal/14-9161-73).
Anti-mouse CD16/32 (93), purified (eBioscience, Clone 93, Cat No: 101302, LOT: B289215, specificity: C57BL/6 mouse splenocytes were stained with purified 93, followed by anti-rat IgGs FITC by the manufacturer, each lot of this antibody is quality control tested by immunofluorescent staining with flow cytometric analysis: https://www.biolegend.com/en-us/global-elements/pdf-popup/purified-anti-mouse-cd16-32-antibody-190?filename=Purified%20anti-mouse%20CD1632%20Antibody.pdf&pdfgen=true).
Anti-human CD5 PE-Cy7 (Bio Legend, clone, Cat No: 364013, LOT: B301091, specificity: Human peripheral blood lymphocytes were stained with CD5 (clone L17F12) PE/Cyanine7 or mouse IgG2a, PE/Cyanine7 isotype control as described by the manufacturer: https://www.biolegend.com/en-us/products/pe-cyanine7-anti-human-cd5-antibody-10479?GroupID=GROUP28).
Anti-human CD19 APC (Bio Legend, clone HIB19, Cat No: 302212, LOT: B245672, specificity: Human peripheral blood lymphocytes were stained with HIB19 APC by the manufacturer: https://www.biolegend.com/en-us/products/apc-anti-human-cd19-antibody-715?GroupID=GROUP28).
Anti-human CD45 PerCP-Cy5.5 (Invitrogen, clone 2D1, Cat No: 45-9459-42, LOT: 4334219, specificity: Staining of normal human peripheral blood cells with Mouse IgG1 K Isotype Control PerCP-Cyanine5-5 or Anti-Human CD45 PerCP-Cyanine5-5 was performed by the manufacturer, in addition several publications, which are cited on the manufactorers website, applied this antibody: https://www.thermofisher.com/antibody/product/CD45-Antibody-clone-2D1-Monoclonal/45-9459-42).
Anti-mouse CD19 APCeFluor780 (eBioscience, clone 1D3, Cat No: 47-0193-82, LOT: 2191655, specificity: the antibody was tested for flow cytometry by the manufacturer using BALB/c splenocytes, in addition numerous publications, which are cited on the manufacturers website used this antibody: https://www.thermofisher.com/antibody/product/CD19-Antibody-clone-eBio1D3-1D3-Monoclonal/47-0193-82).
Anti-mouse CD19 BV510 (BioLegend, clone 6D5, Cat No 115546, LOT: B220306, specificity: C57BL/6 mouse splenocytes were stained with CD19 (clone 6D5) Brilliant Violet 510™ or rat IgG2a, κ Brilliant Violet 510™ control by the manufacturer: https://www.biolegend.com/de-de/products/brilliant-violet-510-anti-mouse-cd19-antibody-8563?GroupID=GROUP20).
Anti-mouse CD45 PE (Invitrogen, clone 30-F11, Cat No: 12-0451-82, LOT: 2051673, specificity: validation was performed by the manufacturer using C57BL/6 mouse bone marrow cells, in addition several publications, cited on the website, applied this antibody: https://www.thermofisher.com/antibody/product/CD45-Antibody-clone-30-F11-Monoclonal/12-0451-82).
Anti-mouse CD5 PE (eBioscience, clone 53-7.3, Cat No: 100608, LOT: B283693, specificity: C57BL/6 mouse splenocytes were stained with 53-7.3 PE for validation by the manufacturer, in addition numerous publications apples this antibody: https://www.biolegend.com/en-us/products/pe-anti-mouse-cd5-antibody-160?GroupID=GROUP20).
Anti-mouse CD5 APC (eBioscience, clone 53-7.3, Cat No: 17-0051-82, specificity: mouse splenocytes were stained with appropriate isotype controls for validation by the manufacturer, in addition the antibody was applied in various publications that are cited on the manufacturers website: https://www.thermofisher.com/antibody/product/CD5-Antibody-clone-53-7-3-Monoclonal/17-0051-82).
Anti-phosho AKT Ser473 PE conjugate (3µl/1e6 cells, CST, clone D9E, Rabbit mAb, Cat No: 5315, specificity: Flow cytometric analysis of Jurkat cells un/treated with LY294002 (PI3 Kinase Inhibitor),Wortmannin (PI3 Kinase Inhibitor) and U0126 (MEK1/2 Inhibitor) using Phospho-Akt (Ser473) (D9E) Rabbit mAb (PE Conjugate) was performed by the manufacturer: https://www.cellsignal.com/products/antibody-conjugates/phospho-akt-ser473-d9e-xp-rabbit-mab-pe-conjugate/5315. In addition we validated specific binding with a matched isotype control and show the P-AKT level of 3AC treated MEC-1 cells compared to untreated control cells.)

If not stated otherwise all flow cytometry antibodies were diluted 1:200.

Western Blot: phospho-Akt Ser473 (Cell Signaling Technology (CST) #9271, Lot 14, specificity: extracts from NIH/3T3 cells, untreated or treated with PDGF, wortmannin, LY294002, rapamycin or PD98059, using Phospho-Akt (Ser473) Antibody as well as extracts from NIH/3T3 cells, untreated or treated with PDGF using Phospho-Akt (Ser473) Antibody or Akt Antibody #9272 were validated by the manufacturer. In addition, Western blot analysis of immunoprecipitated Akt from 293 cells transiently transfected with HA-tagged Akt (WT), HA-tagged K179A mutant Akt and HA-tagged K179A/S473A mutant Akt, using Phospho-Akt (Ser473) Antibody, Akt antibody or HA antibody. Phospho-Akt (Ser473) Antibody does not recognize Akt with an alanine substituion at Ser473. (Polakiewicz, R.D. et al. [1998] J. Biol. Chem. 273, 23534-23541: https://www.cellsignal.com/products/primary-antibodies/phospho-akt-ser473-antibody/9271).
AKT (CST #9272, Lot 22, specificity: validated by the manufacturer as described above and by AKT siRNA experiments in CHO and HeLa cells: https://www.cellsignal.com/products/primary-antibodies/akt-antibody/9272).
phospho-SHIP-1 Tyr1020 (CST #3941, Lot 3, specificity for Tyr1020 of SHIP1 was validated by IgM un/stimulated Ramos cells (human) and IgG un/stimulated mouse splenocytes.: https://www.cellsignal.com/products/primary-antibodies/phospho-ship1-tyr1020-antibody/3941).
SHIP-1 (clone C40G9, CST #2727, Lot 3, specificity: the antibody was validated in RL and Molt4 extracts (https://www.cellsignal.com/products/primary-antibodies/ship1-c40g9-rabbit-mab/2727) and our own studies confirmed the specificity upon SHIP1 knockout and knockdown in MEC-1 cells).
MCL-1 (CST #94296, Lot 1, specificity: various human and rodent cell lines were tested for MCL-1 expression by the manufacturer by Western blot analysis, moreover extracts from 293T cells, mock transfected or transfected with a construct expressing full-length human Mcl-1 or mouse Mcl-1 were analyzed by Western Blot: https://www.cellsignal.com/products/primary-antibodies/mcl-1-d2w9e-rabbit-mab/94296).
SHIP2 (CST #2730, Lot 2, specificity: Western Blot analysis of SKMEL5 and HeLa extracts were performed by the manufacturer: https://www.cellsignal.com/products/primary-antibodies/ship2-antibody/2730).
Tubulin A (clone 11H10, CST, #2125, Lot 11, specificity: Western blot analysis of extracts from C6, COS-7, NIH/3T3 and HeLa cells were performed by the manufacturer: https://www.cellsignal.com/products/primary-antibodies/a-tubulin-11h10-rabbit-mab/2125).
P-S6 Ribosomal protein S235/236 (clone D57.2.2E, CST #4858 Lot: 16, specificity: the manufacturer performed Western blot analysis of extracts from PC12 and NIH/3T3 cells, treated with λ phosphatase, FBS or PDGF and confirmed Phospho-S6 Ribosomal Protein specificity: https://www.cellsignal.com/products/primary-antibodies/phospho-s6-ribosomal-protein-ser235-236-d57-2-2e-xp-rabbit-mab/4858).
HMGB-1 (Abcam ab18256, Lot: GR3237484-1, specificity: The manufacturer tested NIH/3T3, MEF1, PC-12, HeLa, Jurkat, A-431 and HEK-293 whole cell lysates. Besides the specific detection was validated by HMGB-1 knockout in HAP1 whole cell lysates: https://www.abcam.com/hmgb1-antibody-ab18256.html).
DHX9 (Proteintech Europe 17721-1-AP, Cat No: 17721-1-AP, specificity: The antibody was validated by the manufacturer for Western

Blot analysis in Jurkat cells, mouse testis tissue, rat testis tissue and rat spleen tissue: https://www.ptglab.com/products/DHX9-Antibody-17721-1-AP.htm).
ActB (Proteintech Europe, Cat No 66009-I-Ig, 1:60000, specificity: the manufacturer tested various tissues and cell lines by Western Blot and further validated specific binding by knockdown experiments using shActB versus shcontrol in in A549 cells: https://www.ptglab.com/products/Pan-Actin-Antibody-66009-1-Ig.htm).
mouse IgG HRP-linked (horse polyclonal IgG) CST #7076, Lot 29, specificity: according to the manufacturer the antibody is thoroughly validated with CST primary antibodies and will work optimally with the CST western immunoblotting protocol, ensuring accurate and reproducible results: https://www.cellsignal.com/products/secondary-antibodies/anti-mouse-igg-hrp-linked-antibody/7076
rabbit IgG HRP-linked goat polyclonal IgG (CST #7074, Lot 28, specificity: according to the manufacturer the antibody is thoroughly validated with CST primary antibodies and will work optimally with the CST western immunoblotting protocol, ensuring accurate and reproducible results: https://www.cellsignal.com/products/secondary-antibodies/anti-rabbit-igg-hrp-linked-antibody/7074).
ActB antibody was diluted 1:60000, all other primary Western Blot antibodies were diluted 1:1000 and all secondary antibodies were applied at 1:2000.
Histology: CD20cy Antibody Concentrate (diluted 1:2000, clone L26, Agilent, Cat No: M075501-2, specificity: The antibody was clustered as anti-CD20 at the Fifth International Workshop and Conference on Human Leucocyte Differentiation Antigens held in Boston 1993. SDS-PAGE analysis of immunoprecipitates formed between 125I-labeled tonsil cell lysate and the antibody shows reaction primarily with 30 kDa and 33 kDa polypeptides. Studies using COS-1 cells transfected with cDNA encoding the CD20 molecule, indicate that the antibody labels an intracytoplasmic epitope localized on the CD20 molecule. References are provided on the package insert and on the website: https://www.agilent.com/en/product/immunohistochemistry/antibodies-controls/primary-antibodies/cd20cy-(concentrate)-76520).

# Eukaryotic cell lines

Policy information about cell lines

| | |
|---|---|
| Cell line source(s) | Chronic B-cell leukemia-derived cell lines MEC-1 (RRID: CVCL_1870), EHEB (RRID: CVCL_1194) and lymphoma lines SUDHL6 (RRID: CVCL_2206) and BJAB (RRID: CVCL_5711) and Bal17 (RRID:CVCL_9474) and HEK293T cells (RRID:CVCL_0063) were purchased from DSMZ (Braunschweig, Germany). The origin of the H1299 cells (RRID:CVCL_0060) was unknown and therefore authenicated prior to use. |
| Authentication | The MEC-1 and EHEB cell line were authenticated by DSMZ via short tandem repeat (STR)-DNA profiling and the H1299 line via PCR-single-locus-technology (eurofins). The remaining cell lines were not authenicated. |
| Mycoplasma contamination | All cell lines were routinely tested negative for mycoplasma infection. |
| Commonly misidentified lines (See ICLAC register) | No commonly misidentified cell line was used. |

# Animals and other organisms

Policy information about studies involving animals; ARRIVE guidelines recommended for reporting animal research

| | |
|---|---|
| Laboratory animals | The age of the used mice is indicated in the figure legends; if not stated, adult female mice aged 6-12 weeks were used for all experiments; C57BL/6N mice were purchased from Janvier, NSG mice (NOD.Cg-Prkdcscid Il2rgtm1WjI/SzJ) were purchased from Charles River or Janvier (NXG). TCL1-transgenic mice Tg(Igh-V186.2-TCL1A)3Cro were kindly provided by Carlo Croce and CLL harvested when symptoms occured (approx. 8-12 months of age). AKT-E17K mice were generated as a mixed 129X1x129S1xC57BL/6J background and were crossed for at least four generations onto C57BL/6N background. All mice were kept under specific pathogen free condition. |
| Wild animals | The study did not involve wild animals. |
| Field-collected samples | The study did not involve samples collected from the field. |
| Ethics oversight | The Government of Upper Bavaria (Regierung von Oberbayern) approved the study protocol. |

Note that full information on the approval of the study protocol must also be provided in the manuscript.

# Human research participants

Policy information about studies involving human research participants

| | |
|---|---|
| Population characteristics | Peripheral blood was taken after informed consent by CLL patients. All patients were treatment naïve or off CLL therapy for at least 3 months. Healthy donor-derived blood samples (age-matched) were received from the „Bayerisches rotes Kreuz“ (Munich, Germany). |
| Recruitment | Patients were recruited during their routine check-ups at Klinikum Rechts der Isar or München Krankenhaus, Schwabing after given sufficent information about the study by the treating physician. |
| Ethics oversight | The local ethics committee of the Faculty of Medicine, Technical University Munich approved patient sampling and all depicted experiments. |

Note that full information on the approval of the study protocol must also be provided in the manuscript.

# Flow Cytometry

## Plots

Confirm that:

- [x] The axis labels state the marker and fluorochrome used (e.g. CD4-FITC).
- [x] The axis scales are clearly visible. Include numbers along axes only for bottom left plot of group (a 'group' is an analysis of identical markers).
- [x] All plots are contour plots with outliers or pseudocolor plots.
- [x] A numerical value for number of cells or percentage (with statistics) is provided.

## Methodology

| | |
|---|---|
| Sample preparation | Peripheral blood mononuclear cells (PBMCs) were isolated from whole blood by density gradient centrifugation using Ficoll-Paque (GE Healthcare, Chicago, IL, USA). CD19+ B-cells were purified by magnetic-activated cell sorting (MACS) using human B-cell isolation kit II or human CD19 MicroBeads (Miltenyi, Bergisch-Gladbach, Germany). Purification of CLL cells (CD19+ CD5+) was performed using fluorescence-activated cell sorting (FACS) (BD Aria II, BD Bioscience, Franklin Lakes, NJ, USA). Peripheral blood, spleen, and axillary lymph nodes were harvested per mouse. Organs were meshed through a 70 µm cell strainer in PBS buffer and erythrocytes were lysed using G-DEXTMIIb RBC Lysis Buffer (Intron Biotechnologies). Cells were stained with fluorochrome-labeled antibodies to manufacturer information. To block free Fc receptors murine CD16/32 or human Fc Receptor Binding Inhibitor Polyclonal Antibody (eBioscience) were applied. Dead cells were excluded by DAPI (1 µg/ml) (Sigma Aldrich) staining. |
| Instrument | Sorting: FACSAriaTM III, otherwise FACSCantoTM II (BD Biosciences) |
| Software | FlowJo version 10.6.2 |
| Cell population abundance | Reanalysis of post-sort fractions: CD19+CD5+ CLL populations (for RNAseq); GFP+ population or myrAKT and Cas9+ MEC-1 cells. |
| Gating strategy | Gate 1: FSC-A/SSC-A parameters were used for gating on lymphocytes<br>Gate 2: FSC-H/FSC-W parameters were used for gating on singlets.<br>Gate 3: SSC-H/SSC-W parameters were used for gating on singlets.<br>Gate 4: DAPI negative cells were used for gating on living cells |

- [x] Tick this box to confirm that a figure exemplifying the gating strategy is provided in the Supplementary Information.
